# Menopausal hormone therapy and breast cancer risk: a population-based cohort study of 1.3 million women in Norway

**DOI:** 10.1038/s41416-024-02590-1

**Published:** 2024-05-13

**Authors:** Nathalie C. Støer, Siri Vangen, Deependra Singh, Renée T. Fortner, Solveig Hofvind, Giske Ursin, Edoardo Botteri

**Affiliations:** 1 Department of Research, Cancer Registry of Norway, Norwegian Institute of Public Health, Oslo, Norway; 2 Norwegian Research Centre for Women’s Health, Division of Obstetrics and Gynecology, Oslo University Hospital, Oslo, Norway; 3 Institute of Clinical Medicine, University of Oslo, Oslo, Norway; 4 Cancer Surveillance Branch, International Agency for Research on Cancer, WHO, Lyon, France; 5 Division of Cancer Epidemiology, German Cancer Research Center (DKFZ), Heidelberg, Germany; 6 Section for Breast Cancer Screening, Cancer Registry of Norway, Norwegian Institute of Public Health, Oslo, Norway; 7 Department of Health and Care Sciences, UiT The Arctic University of Norway, Tromsø, Norway; 8 Cancer Registry of Norway, Norwegian Institute of Public Health, Oslo, Norway; 9 Department of Nutrition, Institute of Basic Medical Sciences, University of Oslo, Oslo, Norway; 10 Department of Preventive Medicine, University of Southern California, Los Angeles, CA USA; 11 Section for Colorectal Cancer Screening, Cancer Registry of Norway, Norwegian Institute of Public Health, Oslo, Norway

**Keywords:** Risk factors, Breast cancer

## Abstract

**Background:**

It is important to monitor the association between menopausal hormone therapy (HT) use and breast cancer (BC) risk with contemporary estimates, and specifically focus on HT types and new drugs.

**Methods:**

We estimated hazard ratios (HR) of BC risk according to HT type, administration route and individual drugs, overall and stratified by body mass index (BMI), molecular subtype and detection mode, with non-HT use as reference.

**Results:**

We included 1,275,783 women, 45+ years, followed from 2004, for a median of 12.7 years. Oral oestrogen combined with daily progestin was associated with the highest risk of BC (HR 2.42, 95% confidence interval (CI) 2.31–2.54), with drug-specific HRs ranging from Cliovelle®: 1.63 (95% CI 1.35–1.96) to Kliogest®: 2.67 (2.37–3.00). Vaginal oestradiol was not associated with BC risk. HT use was more strongly associated with luminal A cancer (HR 1.97, 95% CI 1.86–2.09) than other molecular subtypes, and more strongly with interval (HR 2.00, 95% CI: 1.85–2.15) than screen-detected (HR 1.40, 95% CI 1.34–1.47) BC in women 50–71 years. HRs for HT use decreased with increasing BMI.

**Conclusions:**

The use of oral and transdermal HT was associated with an increased risk of BC. The associations varied according to HT type, individual drugs, molecular subtype, detection mode and BMI.

## Background

There is convincing evidence that use of menopausal hormone therapy (HT)—both combined oestrogen-progestin therapy (EPT) and unopposed oestrogen therapy (ET)—increases the risk of breast cancer (BC) [1] with increased risk of BC observed even 10 years after cessation. In the wake of this evidence, in 2020 the Pharmacovigilance Risk Assessment Committee of the European Medicines Agency updated its recommendations for product characteristics with a reinforced warning that the use of both ET and EPT increase the risk of BC [2].

When reaching menopause, most women experience menopausal symptoms, which may impair their quality of life. In a study with participants from US, Canada, and Europe, 72% reported hot flushes, 41% fatigue, 10% pain during urination, and 30% reported that the impact of menopause was worse than expected [3]. Other studies have shown that ~12% of women will continue to experience vasomotor symptoms 11–12 years after the last menstrual period [4]. Despite these figures, due to the accumulating knowledge on the health risks associated with the use of HT, prescribing it has become more restrictive.

The risk of BC associated with the use of ET and EPT is well documented, but it is also associated with other adverse health outcomes, such as blood clots and stroke. Still, menopausal HT remains an important medication for mitigating menopausal symptoms, with the added benefit of improving bone health. There have been substantial efforts to develop new preparations with lower risk. With a high number of women in need of treatment for severe menopausal symptoms, detailed contemporary studies are needed to help women and clinicians to choose the best treatment strategy.

In this large population-based cohort study, we provide a detailed picture of the risk of BC according to type of HT, route of administration and individual drugs used. We present results by molecular subtypes, detection mode (i.e., screen-detected and symptomatic cancer), stage at diagnosis, and body mass index (BMI). We also report dose–response analyses for the duration of use and time since the last use.

## Materials and methods

### Data source and study population

All Norwegian residents are assigned an 11-digit unique personal identification number at birth or immigration. The personal identification number allows univocal linkage between national registries. To study the association between the use of HT and the risk of BC, we linked information from national registries and questionnaires. Statistics Norway and the Norwegian Population Registry provided information about date of birth, immigration and emigration status, education, children and region of residence. The date of death was extracted from the Cause of Death Registry. Cancer information was provided by the Cancer Registry of Norway (CRN; including Incidence Database [5], Breast Cancer Registry [6] and the national screening programme for breast cancer, BreastScreen Norway, all administered by the CRN). Information about redeemed prescriptions, including information of date, type classified according to the Anatomical Therapeutic Chemical (ATC) classification system, brand name, strength and total dose, were collected from the Norwegian Prescription Database (NorPD) [7]. BMI was extracted from the Norwegian Regional Health Surveys administered by the Norwegian Institute of Public Health (The Norwegian Counties Study [1974–1988, 1.5% of cohort], the Age 40 Program [1985–1999, 4.1%], Cohort of Norway [1994–2003, 3.6%]) and health indicator questionnaires administered by BreastScreen Norway [2005–2015, 36.2%] [8–10].

The original cohort consisted of all Norwegian residents alive on January 1, 2004, born between 1925 and 1986, and residing in Norway any time from 2004 to 2018. We excluded subjects with an invasive cancer diagnosis (except non-melanoma skin cancer, International Classification of Disease 10^th^ revision (ICD-10) code C44) prior to start of follow-up (*n* = 104,675), subjects with less than 6 months of observation time in the cohort (*n* = 35,595), males (*n* = 1,877,176), women not reaching the age of 45 at the end of follow-up (*n* = 517,635; Supplementary Fig. 1). The final study sample comprised 1,275,783 women.

Women were included in the cohort from January 1, 2004, the month they turned 45 years, or immigrated to Norway, whatever happened latest. Follow-up started 6 months after inclusion in the cohort to ensure at least 6 months of medication history. In the following, date of start of follow-up is referred to as the baseline.

### Nested case–control sample

To study the duration of use and time since last use, we sampled a 1:10 nested case–control study from the cohort for computing efficiency. The controls were matched on the date of inclusion in cohort (plus/minus 6 months) and required to be at risk (i.e., BC-free, alive and residing in Norway) at the age (precise to the month) of BC diagnosis of the case (defined as the index date).

### Exposure definition

Data on HT use was collected from NorPD by retrieving all prescriptions of sex hormones in the ATC-groups G03C (oestrogens) and G03F (oestrogens and progestins in combination), redeemed from 2004 to 2018. NorPD contains individual-level information on all redeemed prescriptions from 2004 and onwards, for the entire Norwegian population, and registration is mandatory by law.

The duration of each HT prescription since 2004 was assumed to be 3 months, as chronically used drugs are usually prescribed for 3 months at a time. Gaps between prescriptions shorter than 4 months (i.e., <7 months between two redeemed prescriptions) were considered as continuous use, whereas longer gaps were assumed to be a stop in use with possible re-uptake. According to their dispensed products, current users were categorised as oestradiol, oestriol, oestradiol combined with norethisterone acetate (oestradiol-NETA), oestradiol combined with medroxyprogesterone acetate (oestradiol-MPA) or tibolone users. Current users were further categorised according to type of combined regimen (continuous: NETA or MPA added to oestrogen daily, or sequential: NETA or MPA added to oestrogen usually for 10–12 days of a cycle), route of administration (oral, transdermal, vaginal) and according to specific drugs used. Women changing from one group to another with a gap shorter than 4 months were defined as mixed users from the date they changed.

In the cohort analyses, women contributed person-years at risk as non-users (e.g., no HT-prescriptions at start of follow-up until the first possible prescription), current users and/or past users. A past user was defined as a woman with more than 7 months since the last redeemed prescription. Person-years at risk within a particular category was calculated from start of study, or the date they entered the category, until BC or censoring, or the date they moved into another category (Supplementary Fig. 2A).

In the nested case–control analyses, duration of use was calculated among current users at index date. Duration of all user periods prior to index date was cumulated according to type and route of administration, and categorised into <1, 1–2.9, 3–4.9, and ≥5 years of use, or unknown duration. Women with any HT prescriptions in the first 5 years after cohort entry were defined as prevalent users. The duration of use for prevalent users was defined as unknown unless their cumulative use was longer than 5 years, and hence defined as ≥5 years. Time since last use was calculated among past users at index date as the number of years between the index date and the end date of the last registered prescription and categorised as <1, 1–2.9, 3–4.9, 5–9.9 and ≥10 years since cessation (Supplementary Fig. 2B).

### Outcome definition

BC diagnoses were retrieved from CRN. Cancer reporting to CRN has been mandatory by law in Norway since 1952. The registry is estimated to be 98.8% complete with 99.3% of BC cases morphologically verified [11].

Invasive BC carcinoma, defined as ICD-10 code C50 and international classification of disease for oncology third revision (ICD-O3) morphology codes 8010–8671 or 8940–8941, was the outcome of interest. The detection mode was categorised as interval cancer (cancer diagnosed between two screening rounds in BreastScreen Norway), screen-detected cancer, and cancer detected outside the screening programme. The recommended screening interval in Norway is 24 months. Screen-detected cancer was defined as breast cancer diagnosed as a result of a positive screening test within 6 months after screening. Interval cancers were defined as breast cancers detected after a negative screening result or more than 6 months after a false positive screening result and within 24 months after screening. Breast cancer detected outside the screening programme was diagnosed among women never invited, invited but did not attend, or detected more than 24 months after last screening examination. Stage was categorised as localised, regionally advanced, and metastatic according to the United States National Cancer Institute’s Surveillance, Epidemiology and End Result Programme.

Molecular subtypes were approximated using the immunohistochemical markers oestrogen receptor (ER) status, progesterone receptor (PR) status and human epidermal growth factor receptor 2 (HER2) expression, in addition to the proliferation index Ki-67 that the CRN registers from pathology reports. Subtypes were defined as luminal A (ER + , PR + , HER2- and low Ki-67), luminal B HER2 negative (ER + , HER2-, PR- and/or high Ki-67), luminal B HER2 positive (ER + , HER2 + , any PR and any Ki-67), HER2 positive (ER−, PR− and HER2 + ) and triple negative (ER−, PR−, HER2−) [12]. Tumours were classified according to the pathology guidelines at the time. Therefore, tumours diagnosed prior to February 2010 were classified as ER- if <10% ER expression, and from February 2010 onwards if <1% ER expression. Tumours were defined as PR- if <10% PR expression, throughout the study period. The cut-off for low/high Ki-67 was set at 14% (low Ki-67 ≤ 14%, high Ki-67 > 14%). When Ki-67 was missing, tumour grade I was used as a proxy for low Ki-67 and tumour grade II-III for high Ki-67 [13].

### Statistical analysis

In the cohort analyses, hazard ratios (HRs) with 95% confidence intervals (CIs) were estimated by Cox proportional hazard models with age as the time scale and time-dependent exposures. Women were censored on December 31, 2018, at the time of another cancer diagnosis (except non-melanoma skin cancer), emigration, first use of hormones other than HT, or death, whichever happened first. HT was analysed according to the use of current and past HT, and current use was further categorised based on type of HT, type of oestradiol-NETA regimen, route of administration and individual drugs. The risk of BC according to the use of HT was analysed overall and stratified by BMI, BC subtype, detection mode and stage at diagnosis. The reference group in all analyses was no prior use of HT.

We adjusted all estimates for ethnicity (Norwegian, other Nordic or non-Nordic), number of children (0, 1, 2, 3 or >3), the highest level of education at baseline (non/mandatory only, secondary, higher education or missing), income at baseline divided into quartiles and missing, region of residence at baseline (South-East, West, Mid, North and missing), time-dependent screening attendance (never, <2.5 years since last screening or ≥2.5 years since last screening), and time-dependent (never/ever) use of antidiabetic medication (A10), antithrombotic agents (B01), antihypertensives (C02), diuretics (C03), beta-blockers (C07), calcium channel blockers (C08), angiotensin-converting enzyme inhibitors and angiotensin receptor blockers (C09), lipid-modifying agents (C10), uterotonics and other gynecologicals (G02), urologicals (G04), thyroid therapy (H03), and treatment of bone diseases (M05). BMI was categorised as (<18.5, 18.5–24.9, 25.0–29.9, 30.0–34.5 or ≥35.0 and missing) and in case of multiple BMI values from different questionnaires, the closest to baseline was chosen. Missing values in adjustment variables were handled with missing categories.

When analysing the association between HT use and BC risk by molecular subtype, detection mode, and stage at diagnosis, only BCs with that specific subtype/ detection mode/stage was analysed as event, and all other BCs were censored at the date of diagnosis. The analysis of the detection mode was furthermore restricted to women of screening age (i.e., follow-up start at age 50, and women are censored at age 71). Heterogeneity between molecular subtypes and detection mode was evaluated by contrast tests [14]. *P* for trend in BMI stratified models were estimated as the interaction term between HT exposure and continuous BMI. Duration of use, time since last use, and time since last use categorised according to the duration of past use were analysed in the nested case control sample with Cox regression stratified by case–control sets (equivalent to conditional logistic regression), estimating HR with 95% CI.

Since adjustment for and stratification by BMI was only possible in a subset of the women, we conducted a sensitivity analysis where we assessed the effects of HT use in this subset in comparison with estimates from the full cohort. We conducted analyses in this subset with and without adjustment for BMI to evaluate the impact of possible confounding by BMI.

In additional sensitivity analyses, we re-analysed HT use among women aged 55 or older at baseline since we lack information about menopausal status, and among women born after 1950 (i.e., younger than 55 at start of follow-up) to have close to complete history of HT use. In the nested case–control sample we furthermore analysed HT-use to check the validity as compared to the full cohort. Finally, we redefined prevalent use in the nested case–control dose–response analysis to only include women aged 50 or older at baseline since the majority of women below 50 years will not have used HT prior to start of follow-up (i.e., the duration of use is not unknown).

All tests were two-sided. Statistical analyses were performed using R version 4.2.1 (http://cran.r-project.org/).

## Results

We followed 1,275,783 Norwegian women for a median of 12.7 years. During follow-up, 454,262 women used HT at any time during follow-up and 33,654 women were diagnosed with BC. Supplementary Table 1 displays the characteristics of the study population. In general, HT users were more often Norwegian, less likely to be nulliparous and more often users of other drugs, especially statins (C10), urological (G04) and thyroid therapy (H03), compared to non-HT users. Oestriol users were older at the start of follow-up and less educated with lower income compared to both other HT users and non-users.

Current use of HT was associated with an increased risk of BC compared to no-HT use (HR 1.45, 95% CI 1.41–1.49, Table 1). Separated by type of HT, risks were highest for current users of oestradiol-NETA (HR 2.23, 95% CI 2.14–2.33) and tibolone (HR 1.72, 95% CI 1.54–1.91). No significant associations were observed for the current use of oral oestriol (HR 1.09, 95% CI 0.95–1.24), and past use of HT (HR 1.03, 95% CI 1.00–1.07).

**Table 1:** Use of menopausal hormone therapy and risk of breast cancer in a cohort of 1,275,783 women followed from 2004 to 2018.

	Cases	Person-years	HR (95% CI)
No use	21,221	9,261,670	Ref.
Current HT	6633	1,760,913	1.45 (1.41–1.49)
Past HT	5800	2,124,645	1.03 (1.00–1.07)
Type of component in oral or transdermal current users^b^			
Oestradiol	577	160,721	1.42 (1.31–1.54)
Oestriol^a^	218	74,174	1.09 (0.95–1.24)
Oestradiol-NETA	2528	461,841	2.23 (2.14–2.33)
Oestradiol-MPA^a^	16	3941	1.63 (1.00–2.65)
Tibolone^a^	340	76,672	1.72 (1.54–1.91)
Type of combined oestradiol-NETA regimen in current users^c^			
Continuous oestradiol-NETA	2011	325,477	2.42 (2.31–2.54)
Sequential oestradiol-NETA	276	87,512	1.46 (1.30–1.65)
Route of administration in oral or transdermal current users^d^			
Oral oestradiol	366	105,151	1.36 (1.22–1.50)
Transdermal oestradiol	186	50,777	1.48 (1.28–1.71)
Oral continuous oestradiol-NETA	1964	314,062	2.45 (2.33–2.57)
Oral sequential oestradiol-NETA	266	84,084	1.47 (1.30–1.66)
Transdermal continuous oestradiol-NETA	34	8649	1.58 (1.13–2.21)
Transdermal sequential oestradiol-NETA	8	2651	1.34 (0.67–2.68)
Type of component in vaginal current users^e^			
Vaginal oestradiol	1948	744,895	0.96 (0.91–1.01)
Vaginal oestriol	53	21,837	0.95 (0.72–1.24)

*HT* hormone therapy, *NETA* norethisterone acetate, *MPA* medroxyprogesterone acetate.

Hazard ratios (HRs) and 95% confidence intervals (CIs) from Cox regression with age as time scale (age-adjusted) and adjusted for ethnicity, number of children, education, income, health region, screening attendance (never, <2.5 years since last screening, ≥2.5 years since last screening), use of antidiabetics (A10), antithrombotic agents (B01), antihypertensives (C02), diuretics (C03), beta-blockers (C07), calcium channel blockers (C08), angiotensin-converting enzyme inhibitors and angiotensin receptor blockers (C09), lipid-modifying agents (C10), uterotonics and other gynecologicals (G02), urologicals (G04), thyroid therapy (H03) and treatment of bone diseases (M05).

^a^Oral formulation.

^b,c,d,e^Estimates for mixed users not shown (^b^922 cases, 205,019 person-years, ^c^total mixed use: 932 cases and 184,740 person-years, mixed use due to switch between continuous and sequential oestradiol-NETA: 241 cases 48,852 person-years, ^d^total mixed use: 1222 cases, 268,396 person-years, mixed use due to switch between oral and transdermal oestradiol: 25 cases, 4793 person-years, mixed due to switch between oestradiol-NETA formulations: 256 cases, 52,395 person-years, ^e^total mixed use: 1190 cases, 263,102 person-years, mixed use due to switch between vaginal oestradiol and oestriol: 31 cases and 11,814 person-years).

Current use of oral continuous oestradiol-NETA was associated with the highest risk of BC (HR 2.42, 95% CI 2.31–2.54). Split into specific drugs, the risk varied from 63% increased risk among current users of Cliovelle® (HR 1.63, 95% CI 1.35–1.96) to 167% increased risk among current users of Kliogest® (HR 2.67, 95% CI 2.37–3.00, Table 2).

**Table 2:** Use of menopausal hormone therapy preparations and risk of breast cancer in a cohort of 1,275,783 women followed from 2004 to 2018.

	Strength	Mean (minimum, maximum) duration in years^a^	Marketing date	Cases	Person-years	HR (95% CI)
No use				21,221	9,261,670	Ref.
Current oral oestradiol						
Progynova®	1 mg oestradiol/day	2.8 (<0.1–14.5)	01.01.2001–now	140	38,319	1.39 (1.18–1.64)
Progynova®	2 mg oestradiol/day	3.1 (<0.1–14.5)	01.01.2001–now	162	46,378	1.40 (1.20–1.63)
Current oral oestriol						
Ovesterin®	1 mg oestriol/day	2.3 (<0.1–14.5)	01.01.2001–now	100	36,853	1.01 (0.83–1.23)
Ovesterin®	2 mg oestriol/day	1.9 (<0.1–14.5)	01.01.2001–now	45	12,559	1.34 (1.00–1.79)
Current oral continuous oestradiol-NETA						
Eviana®	0.5 mg oestradiol/day; 2.8 mg NETA/month	1.8 (<0.1–9.6)	01.05.2009–now	72	16,922	1.66 (1.32–2.09)
Activelle®	1 mg oestradiol /day; 14 mg NETA/month	2.4 (<0.1–14.5)	13.05.2005–now	957	155,051	2.42 (2.26–2.58)
Cliovelle®	1 mg oestradiol /day; 14 mg NETA/month	1.5 (<0.1–8.5)	01.05.2010–now	112	27,602	1.63 (1.35–1.96)
Kliogest®	2 mg oestradiol/day; 28 mg NETA/month	2.0 (<0.1–11.1)	01.01.2001–10.01.2013	286	42,499	2.67 (2.37–3.00)
Current oral sequential oestradiol-NETA						
Trisekvens®/Trisekvens forte®	2/4 mg oestradiol /day; 10/10 mg NETA/month	1.7 (<0.1–14.5)	01.01.2001–now/ discontinued in 2004	156	45,866	1.57 (1.34–1.83)
Novofem®	1 mg oestradiol /day; 12 mg NETA/month	1.6 (<0.1–14.5)	01.02.2002–now	84	31,936	1.23 (0.99–1.53)
Current oral continuous oestradiol-MPA						
Indivina®	1–2 mg estriol/day; 2.8–5 mg MPA/month	2.6 (<0.1–14.5)	01.03.2001–now	16	3935	1.63 (1.00–2.66)
Current transdermal oestradiol						
Estradot®	0.025–0.100 mg oestradiol/day	2.1 (<0.1–14.5)	01.05.2002–now	127	35,643	1.44 (1.21–1.72)
Evorel®	0.025–0.100 mg oestradiol/day	1.9 (<0.1–11.1)	01.01.2001–31.08.2014	15	4909	1.24 (0.75–2.06)
Current transdermal continuous oestradiol-NETA						
Estalis®	0.05 mg oestradiol /day; 7 mg NETA/month	1.4 (<0.1–14.5)	01.03.2005–04.03.2011	34	8649	1.57 (1.12–2.20)
Current transdermal sequential oestradiol-NETA						
Sequidot®	0.05 mg oestradiol /day; 3.5 mg NETA/month	1.0 (<0.1–7.5)	15.07.2008–now	7	1164	2.62 (1.25–5.50)
Current vaginal oestradiol						
Vagifem® vaginal inserts	0.010 mg oestradiol/day	2.1 (<0.1–8.0)	01.11.2010–now	1059	398,208	0.97 (0.91–1.03)
Vagifem® vaginal inserts	0.025 mg oestradiol/day	2.0 (<0.1–8.2)	01.01.2001–31.10.2011	671	266,187	0.96 (0.89–1.04)
Current vaginal oestriol						
Ovesterin® vaginal inserts	0.5 mg oestriol/day	0.8 (<0.1–14.4)	01.01.2001–now	31	13,854	0.88 (0.62–1.26)
Ovesterin® vaginal cream	1 mg oestriol/1 g cream	0.7 (<0.1–13.1)	01.01.2001–now	20	6924	1.13 (0.73–1.76)

*NETA* norethisterone acetate, *MPA* medroxyprogesterone acetate.

Hazard ratios (HRs) and 95% confidence intervals (CIs) from Cox regression with age as time scale (age-adjusted) and additionally adjusted for ethnicity, number of children, education, income, health region, screening attendance (never, <2.5 years since last screening, ≥2.5 years since last screening), use of antidiabetics (A10), antithrombotic agents (B01), antihypertensives (C02), diuretics (C03), beta-blockers (C07), calcium channel blockers (C08), angiotensin-converting enzyme inhibitors and angiotensin receptor blockers (C09), lipid-modifying agents (C10), uterotonics and other gynecologicals (G02), urologicals (G04), thyroid therapy (H03) and treatment of bone diseases (M05).

^a^Mean, minimum and maximum refer to the observed duration of use after July 2004 and therefore underestimated for all products available prior to 2004. Estimates for mixed use not shown (2199 cases and 490,785 person-years).

Current use of oral (overall: HR 1.36, 95% CI 1.22–1.50; Progynova® 1 mg: HR 1.39, 95% CI 1.18–1.64; Progynova® 2 mg: 1.40, 95% CI 1.20–1.63) and transdermal (overall: HR 1.48, 95% CI 1.28–1.71; Estradot®: HR 1.44, 95% CI 1.21–1.72; Evorel®: HR 1.24, 95% CI 0.75–2.06) oestradiol were associated with increased risk of BC, while no association was observed among users of vaginal oestradiol or oestriol (Tables 1 and 2).

Use of HT was more strongly associated with an increased BC risk in women with low BMI than in women with high BMI (Table 3). Linear trends in BC risk according to BMI were observed for current use of HT, oestradiol-NETA overall, oral oestradiol-NETA (*P*_trends_ <0.001) and transdermal oestradiol-NETA (*P*_trend_ = 0.035), oral oestradiol (*P*_trend =_ 0.011) and tibolone (*P* = 0.021).

**Table 3:** Use of menopausal hormone therapy and risk of breast cancer stratified by body mass index among 579,247 women with available body mass index.

	BMI < 18.5		BMI 18.5–24.9		BMI 25.0–29.9		BMI 30.0–34.9		BMI ≥ 35.0		*P*_trend_
	Cases	HR (95% CI)	Cases	HR (95% CI)	Cases	HR (95% CI)	Cases	HR (95% CI)	Cases	HR (95% CI)	
No use	86	Ref.	4320	Ref.	3596	Ref.	1283	Ref.	466	Ref.	
Current HT	41	1.89 (1.29–2.77)	2249	1.55 (1.47–1.63)	1425	1.30 (1.22–1.38)	362	1.22 (1.08–1.37)	82	1.04 (0.82–1.32)	0.001
Past HT	34	1.22 (0.81–1.84)	1756	1.10 (1.04–1.17)	1411	1.03 (0.97–1.10)	466	1.09 (0.97–1.21)	140	1.06 (0.87–1.29)	0.227
Type of component in oral or transdermal current users^b^											
Oestradiol	3		185	1.59 (1.37–1.84)	127	1.35 (1.13–1.61)	29	1.03 (0.71–1.49)	5	0.62 (0.26–1.50)	<0.001
Oestriol^a^	0		36	1.26 (0.91–1.75)	30	1.07 (0.75–1.54)	10	0.99 (0.53–1.84)	2		0.414
Oestradiol-NETA	23	3.19 (2.00–5.09)	917	2.43 (2.26–2.61)	473	1.90 (1.72–2.09)	109	1.78 (1.46–2.16)	29	1.88 (1.29–2.74)	<0.001
Tibolone^a^	3		123	1.70 (1.42–2.03)	54	1.20 (0.92–1.58)	13	1.15 (0.67–1.99)	2		0.021
Route of administration in oral or transdermal current users^c^											
Oral oestradiol	0		117	1.55 (1.29–1.86)	80	1.22 (0.97–1.52)	19	0.94 (0.59–1.47)	4		0.011
Transdermal oestradiol	3		58	1.54 (1.19–2.00)	41	1.58 (1.16–2.15)	10	1.40 (0.75–2.60)	0		0.095
Oral oestradiol-NETA	20	3.22 (1.97–5.28)	794	2.45 (2.27–2.64)	414	1.91 (1.73–2.12)	91	1.68 (1.35–2.07)	23	1.70 (1.12–2.59)	<0.001
Transdermal oestradiol-NETA	0		13	1.40 (0.81 – 2.41)	9	1.64 (0.85–3.16)	5	4.55 (1.89–10.96)	1		0.035
Type of component in vaginal current users^d^											
Vaginal oestradiol	9	1.07 (0.53–2.15)	621	0.93 (0.85–1.01)	516	0.95 (0.87–1.05)	139	0.92 (0.77–1.10)	38	0.92 (0.66–1.29)	0.058
Vaginal oestriol	0		10	0.91 (0.49–1.70)	7	0.66 (0.31–1.38)	8	1.92 (0.95–3.84)	0		0.601

*BMI* body mass index, *HT* hormone therapy, *NETA* norethisterone acetate.

Hazard ratios (HRs) and 95% confidence intervals (CIs) from Cox regression with age as time scale (age-adjusted) and additionally adjusted for ethnicity, number of children, education, income, health region, screening attendance (never, <2.5 years since last screening, ≥2.5 years since last screening), use of antidiabetics (A10), antithrombotic agents (B01), antihypertensives (C02), diuretics (C03), beta-blockers (C07), calcium channel blockers (C08), angiotensin-converting enzyme inhibitors and angiotensin receptor blockers (C09), lipid-modifying agents (C10), uterotonics and other gynecologicals (G02), urologicals (G04), thyroid therapy (H03) and treatment of bone diseases (M05).

^a^Oral formulation.

^b,c,d^Estimates for mixed use not shown (^b^BMI < 18.5: 3 cases, BMI 18.5–24.9: 341 cases. BMI 25.0–29.9: 206 cases, BMI 30.0–34.9: 52 cases, BMI ≥ 35: 6 cases, ^c^BMI < 18.5: total mixed use: 6 cases, mixed use due to switch between oral and transdermal oestradiol-NETA: 3 cases, BMI 18.5–24.9: total mixed use: 467 cases, mixed use due to switch between oral and transdermal oestradiol: 10, mixed use due to switch between oral and transdermal oestradiol-NETA: 110, BMI 25.0–29.9: total mixed use: 265 cases, mixed use due to switch between oral and transdermal oestradiol: 6 cases, mixed use due to switch between oral and transdermal oestradiol-NETA: 50 cases, BMI 30.0–34.9: total mixed use: 67 cases, mixed use due to switch between oral and transdermal oestradiol: 0 cases, mixed use due to switch between oral and transdermal oestradiol-NETA: 13 cases, BMI ≥ 35.0**:** total mixed use**:** 12 cases, mixed use due to switch between oral and transdermal oestradiol: 1 case, mixed use due to switch between oral and transdermal oestradiol-NETA: 5 cases, ^d^BMI < 18.5: total mixed use: 6 cases, mixed use due to switch between vaginal oestradiol and estriol: 0, BMI 18.5–24.9: total mixed use: 453 cases, mixed use due to switch between vaginal oestradiol and oestriol: 8 cases, BMI 25.0–29.9: total mixed use 257, mixed use due to switch between vaginal oestradiol and oestriol: 6 cases, BMI 30.0–34.9: total mixed use: 66 cases, mixed use due to switch between vaginal oestradiol and oestriol: 2 cases, BMI ≥ 35.0: total mixed use: 11 cases, mixed use due switch between vaginal oestradiol and oestriol: 0 cases).

Compared to HT non-users, current users of HT had the highest risk of luminal A (HR 1.97, 95% CI 1.86–2.09) followed by luminal B HER2 negative (HR 1.35, 95% CI 1.29–1.41) and luminal B HER2-positive BC (HR 1.28, 95% CI 1.15–1.43), however current HT users were also at an increased risk of triple-negative BC (HR 1.14, 95% CI 1.02–1.28; *P*_heterogeneity_ < 0.001; Table 4). A similar pattern was observed for oestradiol-NETA (*P*_heterogeneity_ overall <0.001, oral <0.001).

**Table 4:** Use of menopausal hormone therapy and risk of breast cancer stratified by molecular subtypes.

	Luminal A		Luminal B HER2 negative		Luminal B HER2 positive		HER2 positive		Triple negative		*P*_heterogeneity_
	Cases	HR (95% CI)	Cases	HR (95% CI)	Cases	HR (95% CI)	Cases	HR (95% CI)	Cases	HR (95% CI)	
No use	3934	Ref.	9301	Ref.	1808	Ref.	794	Ref.	1499	Ref.	
Current HT	1839	1.97 (1.86–2.09)	2714	1.35 (1.29–1.41)	469	1.28 (1.15–1.43)	192	1.10 (0.94–1.30)	376	1.14 (1.02–1.28)	<0.001
Past HT	1340	1.18 (1.11–1.26)	2658	1.07 (1.02–1.12)	504	1.17 (1.06–1.30)	249	1.20 (1.03–1.39)	440	1.07 (0.96–1.19)	0.080
Type of component in oral or transdermal current users^b^											
Oestradiol	170	2.05 (1.76–2.39)	216	1.18 (1.03–1.35)	52	1.50 (1.13–1.97)	22	1.39 (0.91–2.13)	32	1.11 (0.78–1.58)	<0.001
Oestriol^a^	30	0.85 (0.59–1.21)	65	0.78 (0.61–1.00)	12	0.90 (0.51–1.59)	6	0.96 (0.43–2.15)	18	1.28 (0.80–2.04)	0.483
Oestradiol-NETA	808	3.55 (3.28–3.83)	943	1.88 (1.75–2.01)	161	1.66 (1.41–1.95)	40	0.88 (0.64–1.22)	108	1.32 (1.09–1.61)	<0.001
Tibolone^a^	78	1.88 (1.50– 2.36)	140	1.59 (1.34–1.88)	23	1.40 (0.93–2.11)	9	1.15 (0.59–2.22)	21	1.47 (0.96–2.27)	0.477
Route of administration in oral or transdermal current users^c^											
Oral oestradiol	98	1.78 (1.46–2.18)	138	1.15 (0.97–1.36)	37	1.64 (1.18–2.27)	17	1.64 (1.01–2.66)	20	1.04 (0.67–1.62)	0.008
Transdermal oestradiol	61	2.38 (1.85–3.07)	70	1.21 (0.96–1.53)	11	0.99 (0.55–1.79)	5	1.00 (0.41–2.41)	11	1.24 (0.68–2.25)	<0.001
Oral oestradiol-NETA	705	3.55 (3.27–3.85)	829	1.90 (1.77–2.04)	138	1.65 (1.38–1.96)	36	0.92 (0.66–1.29)	91	1.29 (1.04–1.60)	<0.001
Transdermal oestradiol-NETA	11	1.97 (1.09–3.56)	21	1.68 (1.10–2.58)	2		2		1		0.676
Type of component in vaginal current users^d^											
Vaginal oestradiol	438	1.02 (0.92–1.13)	929	1.04 (0.97–1.12)	141	0.91 (0.76–1.09)	83	1.11 (0.88–1.40)	147	1.01 (0.85–1.21)	0.649
Vaginal oestriol	8	0.78 (0.39–1.57)	21	0.89 (0.58–1.36)	8	1.99 (0.99–3.98)	2		4		0.106

*HER2* human epidermal growth factor receptor 2, *HT* hormone therapy, *NETA* norethisterone acetate.

Hazard ratios (HRs) and 95% confidence intervals (CIs) from Cox regression with age as time scale (age-adjusted) and additionally adjusted for ethnicity, number of children, education, income, health region, screening attendance (never, <2.5 years since last screening, ≥2.5 years since last screening), use of antidiabetics (A10), antithrombotic agents (B01), antihypertensives (C02), diuretics (C03), beta-blockers (C07), calcium channel blockers (C08), angiotensin-converting enzyme inhibitors and angiotensin receptor blockers (C09), lipid-modifying agents (C10), uterotonics and other gynecologicals (G02), urologicals (G04), thyroid therapy (H03) and treatment of bone diseases (M05).

^a^Oral formulation.

^b,c,d^Estimates for mixed use not shown (^b^Luminal A: 293 cases, Luminal B HER2 negative: 380 cases, Luminal B HER2 positive: 69 cases, HER2 positive: 26 cases, triple negative: 44 cases, ^c^Luminal A: total mixed use: 398 cases, mixed use due to switch between oral and transdermal oestradiol: 11 cases, mixed use due to switch between oral and transdermal oestradiol-NETA: 92 cases, Luminal B HER2 negative: total mixed use: 492 cases, mixed use due to switch between oral and transdermal oestradiol: 8 cases, mixed use due to switch between oral and transdermal oestradiol-NETA: 93 cases, Luminal B HER2 positive: total mixed use: 97 cases, mixed use due to switch between oral and transdermal oestradiol: 4 cases, mixed use due to switch between oral and transdermal oestradiol-NETA: 21 cases, HER2 positive: total mixed use: 30 cases, mixed use due to switch between oral and transdermal oestradiol: 0 cases, mixed use due to switch between oral and transdermal oestradiol-NETA: 2 cases, triple negative: total mixed use: 63 cases, mixed use due to switch between oral and transdermal oestradiol: 1 case, switch between oral and transdermal oestradiol-NETA: 16 cases, ^d^Luminal A: total mixed use: 382 cases, mixed use due to switch between vaginal oestradiol and oestriol: 4 cases, Luminal B HER2 negative: total mixed use: 481 cases, mixed use due to switch between vaginal oestradiol and oestriol: 16 cases, Luminal B HER2 positive: total mixed use: 97 cases, mixed use due to switch between vaginal oestradiol and oestriol 3 cases, HER2 positive: total mixed use: 30 cases, mixed use due to switch between vaginal oestradiol and oestriol: 3 cases, triple negative: total mixed use: 62 cases, mixed use due to switch between vaginal oestradiol and oestriol: 2 cases).

Compared to HT non-users, current users of HT had a higher risk of interval cancer (HR 2.00, 95% CI 1.85–2.15) than screen-detected BC (HR 1.40, 95% CI 1.34–1.47), or cancer detected outside the screening programme (HR 1.30, 95% CI 1.20–1.39; *P*_heterogeneity_ < 0.001; Table 5). A similar pattern was observed across all exposure categories, with significant heterogeneity for past HT use (*P*_heterogeneity_ <0.001), and current use of oestradiol (*P*_heterogeneity_ overall <0.001, oral <0.001), oestradiol-NETA (*P*_heterogeneity_ overall <0.001, oral <0.001) and tibolone (*P*_heterogeneity_ < 0.001).

**Table 5:** Use of menopausal hormone therapy and risk of breast cancer stratified by detection mode among 925,874 women aged 50-71 (women eligible for mammographic screening with two years of additional follow-up).

	Interval cancer		Screening detected cancer		Detected outside of screening		*P*_heterogeneity_
	Cases	HR (95% CI)	Cases	HR (95% CI)	Cases	HR (95% CI)	
No use	2,036	Ref.	7,073	Ref.	3,035	Ref.	
Current HT	1,206	2.00 (1.85 – 2.15)	2,857	1.40 (1.34 – 1.47)	1,064	1.30 (1.20 – 1.39)	<0.001
Past HT	817	1.24 (1.14 – 1.35)	2,361	1.02 (0.98 – 1.08)	849	0.90 (0.83 – 0.97)	<0.001
Type of component in oral or transdermal current users^b^							
Oestradiol	137	2.49 (2.09 – 2.96)	216	1.16 (1.00 – 1.32)	85	1.12 (0.91 – 1.39)	<0.001
Oestriol^a^	15	1.25 (0.80 – 2.08)	41	0.93 (0.68 – 1.26)	19	0.95 (0.60 – 1.49)	0.606
Oestradiol-NETA	478	2.89 (2.61 – 3.19)	1,181	2.16 (2.03 – 2.30)	453	2.01 (1.82 – 2.22)	<0.001
Tibolone^a^	78	2.50 (2.00 – 3.14)	141	1.39 (1.18 – 1.65)	79	1.93 (1.55 – 2.42)	<0.001
Route of administration in oral or transdermal current users^c^							
Oral oestradiol	88	2.46 (1.99 – 3.05)	133	1.07 (0.90 – 1.28)	53	1.05 (0.80 – 1.37)	<0.001
Transdermal oestradiol	43	2.41 (1.78 – 3.26)	74	1.28 (1.01 – 1.60)	27	1.17 (0.80 – 1.71)	0.002
Oral oestradiol-NETA	410	2.89 (2.60 – 3.22)	1,049	2.21 (2.07 – 2.36)	387	1.98 (1.78 – 2.21)	<0.001
Transdermal oestradiol-NETA	8	1.91 (0.95 – 3.83)	25	1.91 (1.29 – 2.83)	4		1.000
Type of component in vaginal current users^d^							
Vaginal oestradiol	309	1.18 (1.04 – 1.33)	853	0.96 (0.89 – 1.03)	250	0.71 (0.62 – 0.81)	<0.001
Vaginal oestriol	6	1.26 (0.57 – 2.82)	15	0.89 (0.54 – 1.48)	2		0.470

Hazard ratios (HRs) and 95% confidence intervals (CIs) from Cox regression with age as time scale (age adjusted) and additionally adjusted for ethnicity, number of children, education, income, health region, screening attendance (never, <2.5 years since last screening, ≥2.5 years since last screening), use of antidiabetics (A10), antithrombotic agents (B01), antihypertensives (C02), diuretics (C03), beta-blockers (C07), calcium channel blockers (C08), angiotensin-converting enzyme inhibitors and angiotensin receptor blockers (C09), lipid modifying agents (C10), uterotonics and other gynecologicals (G02), urologicals (G04), thyroid therapy (H03) and treatment of bone diseases (M05). HT: hormone therapy; NETA: norethisterone acetate. ^b,c,d^Estimates for mixed use not shown (^b^Interval cancer: 177 cases, screening detected cancer: 395 cases, detected outside of screening: 169 cases ^c^Interval cancer: total mixed use: 248 cases, mixed use due to switch between oral and transdermal oestradiol 6, mixed use due to switch between oral and transdermal oestradiol-NETA: 60, screening detected cancer: total mixed use 512 cases, mixed use due to switch between oral and transdermal oestradiol 9 cases, mixed use due to switch between oral and transdermal oestradiol-NETA 107 cases, detected outside of screening: total mixed use: 240 cases, mixed use due to switch between oral and transdermal oestradiol: 5 cases, mixed use due to switch between oral and transdermal oestradiol-NETA: 62 cases, ^d^ Interval cancer: total mixed use: 240 cases, mixed use due to switch between vaginal oestradiol and oestriol: 5 cases, screening detected cancer: total mixed use: 495 cases, mixed use due to switch between vaginal oestradiol and oestriol: 5 cases, detected outside of screening: total mixed use: 232 cases, mixed use due switch between vaginal oestradiol and oestriol: 4 cases).

**Fig. 1:**
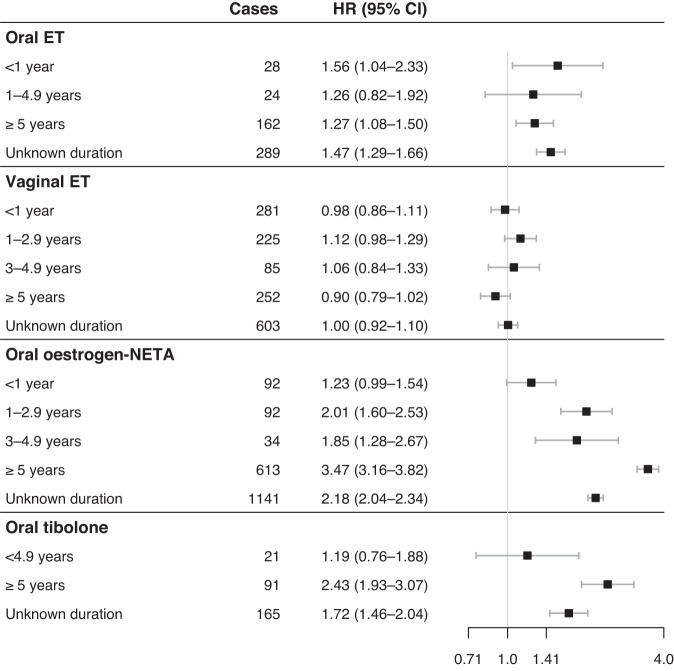
Use of menopausal hormone therapy and risk of breast cancer according to the duration of use in current users compared to non-users in a 1:10 nested case–control sample. Hazard ratios (HRs) and 95% confidence intervals (CIs) from stratified Cox regression with age as time scale (age-adjusted) and additionally adjusted for ethnicity, number of children, education, income, health region, screening attendance (never, <2.5 years since last screening, ≥2.5 years since last screening), use of antidiabetics (A10), antithrombotic agents (B01), antihypertensives (C02), diuretics (C03), beta-blockers (C07), calcium channel blockers (C08), angiotensin-converting enzyme inhibitors and angiotensin receptor blockers (C09), lipid-modifying agents (C10), uterotonics and other gynecologicals (G02), urologicals (G04), thyroid therapy (H03) and treatment of bone diseases (M05). Estimates for mixed use not shown. *ET* oestrogen, *NETA* norethisterone acetate.

In the nested case–control sample, analyses of the duration of use of oral oestradiol-NETA yielded hazard ratios ranging from 1.23 (95% CI 0.99–1.54) among women using it for less than 1 year to 3.47 (95% CI 3.16–3.82) among women using it for more than 5 years (Fig. 1), relative to non-users. No clear trends were observed for time since the last use (Supplementary Fig. 3) or time since the last use in categories of duration of past use.

Compared to HT non-users, current HT users had an increased risk of localised and regionally advanced BC, and users of oestradiol-NETA was also at increased risk of metastatic BC (Supplementary Table 2).

In sensitivity analyses, the risk estimates reported in Table 1 did not change when we additionally adjusted for BMI (Supplementary Table 3) or use of other hormones (ATC code G03 excluding G03C and G03F). When the analyses in Table 1 were repeated for women followed from age 55 or older, the conclusions remained the same, although with increased hazard ratios for all exposures (Supplementary Table 4). Among women born after 1950 the associations were weakened, but the conclusion remained the same. In the nested case–control sample, we repeated the main analyses from Table 1 to evaluate the validity of the sample. The nested case–control estimates were similar to the cohort estimates, except for oestriol use which showed a stronger association with BC risk in the nested case–control sample than in the cohort (Supplementary Table 5). In the dose–response analyses when prevalent users only included women 50 or older, the results were similar. Supplementary Table 6 shows the risk associated with use of individual drugs, without combining doses for Estradot®, Evorel® and Indivina®.

## Discussion

In this Norwegian nationwide population-based cohort, with detailed prescription-based information on menopausal HT use from 2004 to 2018, we found that HT users had a marked increased risk of BC. The risk was highest among oral oestradiol-NETA users with risk increases ranging from 23% with less than 1 year of use to a 3.5-fold increase with ≥5 years of use. Women using tibolone, or oral or transdermal oestradiol, were also at a higher risk of BC, while no association was observed among vaginal oestrogen users. The increased risk of BC in HT users was highest among women with low BMI. HT use was more strongly associated with luminal A BC than other molecular subtypes, and with interval cancer compared to screen-detected cancer.

Overall, our results are in agreement with the well-established increased risk of BC with use of HT reported in observational studies [1]. The increased risk associated with use of EPT observed in our study is of similar size as reported in other European observational studies but higher than estimates from American observational studies [15] and a recent follow-up of the Women’s Health Initiative (WHI) randomised trial [16]. In Norway, and the other Nordic countries, NETA is almost exclusively used as the progestin in EPT, while in other countries, including the U.S., MPA is more commonly used [17]. It has been speculated that this could be the reason for the different risk estimates associated with EPT use across studies [15]. We found a lower increased risk associated with use of oestradiol-MPA than oestradiol-NETA, although the number of oestradiol-MPA users was too small to draw any strong conclusion. Lyytinen et al. [18]. also reported significantly higher BC incidence among women using oestradiol-NETA than oestradiol-MPA, when used for more than 5 years. However, the 2019 meta-analysis of observational studies conducted by the Collaborative Group did not find any difference in risk between the use of oestradiol-MPA and oestrogen-NETA [1]. Difference in monthly dose of progestin has been offered as an alternative explanation for the different risk estimates across studies [19].

Use of oral oestradiol was associated with a 36% increased risk of BC, which is in line with the 38% increased risk reported by the Collaborative Group meta-analysis [1]. It is, however, in strong contrast to the follow-up study of the WHI randomised trial reporting a 22% decreased risk of BC among users of oestrogen [16]. The WHI results have been subject to much debate [20]. The major criticism has been that the age at HT initiation, 64 years on average, is long after menopause when women normally would start using HT. This gap between menopause and HT initiation has been suggested as an explanation for the decreased risk of BC among HT users in the WHI trial. One theory is that such a gap, or oestrogen deprivation period, can cause breast cancer cells to reconfigure; and that a sudden rise in oestrogen levels again, deriving from HT initiation, would simply counteract cell growth by enhancing apoptosis [21].

The use of transdermal HT, especially transdermal oestrogen, is increasing in Norway (Supplementary Fig. 4). Serum concentrations of oestradiol and progesterone have been shown to be similar between the available transdermal and oral formulations of HT [22]. We found similar breast cancer risk estimates for oral and transdermal oestradiol use, which agrees with previous observational studies [1, 19, 23].

The use of tibolone has been steadily declining in Norway since it was first introduced in 2000 (Supplementary Fig. 4). It is a synthetic compound with oestrogenic, androgenic and progestogenic activity. Tibolone does not increase cell proliferation and reduces the level of active oestrogen in the breast [24]. It was therefore promoted as a less risky option than other oestrogen therapies. However, in agreement with previous studies [1, 19, 25], use of tibolone compared to non-HT use was associated with a substantial increased risk of BC.

During the study period, four different oral continuous EPT products were available in Norway. Kliogest® (2 mg oestradiol/day, 28 mg NETA/month) was removed from the market in 2008, Activelle® (1 mg oestradiol/day, 14 mg NETA/month) has been available for the entire study period, while Eviana® (0.5 mg oestradiol/day, 2.8 mg NETA/month) and Cliovelle® (1 mg oestradiol/day, 14 mg NETA/month) entered the market in 2009 and 2010, respectively. Use of Activelle® was associated with a 2.4-fold and Kliogest® a 2.7-fold increased risk compared to non-HT use. The similarity of these two estimates is surprising given that the dose of both oestradiol and NETA in Kliogest® is twice the dose in Activelle®. A potential explanation is that a portion of the Activelle® users had previously used Kliogest®, which could contribute to an artificially high risk associated with use of Activelle®. To test this, we split Activelle® use into Activelle® with no known prior use of Kliogest® and Activelle® with prior use of Kliogest®. Among women using Activelle® with prior use of Kliogest®, the HR increased from 2.42 to 2.90, indicating that prior use of Kliogest® has likely to some extent inflated the risk estimate of Activelle®, reducing the difference between the two drugs. In support of this potential explanation a previous Norwegian study with follow-up from 2004 to 2008 (the period when Kliogest was on the marked), reported larger difference in risk between use of Kliogest® and Activelle® [19].

The dose of oestradiol and NETA in Activelle® and Cliovelle® is identical. Still, we found that use of Cliovelle® (HR 1.63) compared to non-HT use was associated with a significantly (*P* < 0.001) lower risk of BC than use of Activelle® (HR 2.42). This could indicate that the newer drug Cliovelle® is a safer option than Activelle®. Cliovelle® contains oestradiol valerate, while Activelle® contains oestradiol hemihydrate. However, since serum oestradiol levels have been shown to be similar after use of equal dose of oestradiol valerate and oestradiol hemihydrate [26], and Activelle® and Cliovelle® contains the same amount of NETA, the risk difference between them could have other explanations. One potential explanation is the inflation of risk estimates from prior use of Kliogest®, that may have affected Activelle® to a larger degree than Cliovelle®. Since Kliogest® was removed from the market 2 years before Cliovelle® was approved, Kliogest® users could only switch to Activelle® at the time of removal. Thus, more Activelle® than Cliovelle® users have likely used Kliogest® in the past. Another potential explanation is the difference in duration of use. The complete duration of Activelle® use cannot be calculated from our data, as we do not have any information about use prior to 2004, but a conservative estimate, based on cumulating all observed user periods of Activelle® and Cliovelle®, is that Activelle® has been used on average 1 year longer than Cliovelle®. Finally, potential differences in downstream oestrogen metabolites might also play a role.

High BMI is a risk factor for post-menopausal BC [27], and is also associated with higher oestrogen levels in post-menopausal women, as endogenous oestrogen synthesis occurs mainly in adipose tissue after menopause. The increased risk of post-menopausal BC associated with increasing BMI has been shown to be predominantly mediated through oestrogen levels [28]. Additional adjustment for BMI did not impact the association between HT use and BC risk in our study, but we did observe that use of HT was more strongly associated with increased BC risk in women with low BMI than in women with high BMI. The latter is in line with previous studies [1, 29].

As expected, use of HT was more strongly associated with risk of luminal cancers, especially luminal A, than HER2 positive and triple-negative BC. This is in line with several studies showing stronger association with risk of hormone receptor positive, than hormone receptor-negative BC [30–32]. However, more surprisingly, we also observed an increased risk of triple-negative BC among HT users. Interestingly, increased risk of triple-negative BC [33] and ER-/PR- BC [34, 35] have also been observed among oral contraceptive users. One potential explanation is that progestin can interact with other receptors. NETA has some affinity for the androgen receptor [36] which have been shown to promote epithelial–mesenchymal transition, migration and invasiveness in positive triple-negative BC cell lines [37]. Progestin can also interact through progestin-induced paracrine signalling via the receptor activator of nuclear factor Kappa B (RANK) ligand (RANKL). RANKL may in turn bind to RANK on neighbouring breast cells, stimulating proliferation [38]. Another potential explanation is that triple-negative BC cells may still express a certain amount of ER and PR given the defined cut-offs for negative receptor status (<10% PR expression; <10% ERα expression until February 2010 and <1% after February 2010). To test this, we re-analysed the association between HT use and risk of triple-negative BC with start of follow-up in February 2010 to only include triple-negative BC with <1% ERα expression, however the associations remained (results not shown). It is still possible, however, that there was some hormone receptor expression among the triple-negative cancers in our study, given the known heterogeneity of triple-negative cancers [39]. It has also been shown that up to 65% of triple-negative BC express ERβ and up to 70% express the G-protein-coupled oestrogen receptor, thus oestrogen might still have direct growth-stimulating effects in triple-negative BC [38].

The reason behind the higher risk of interval cancer than screen-detected cancer among HT users is not fully understood. One possible explanation is that HT increases mammographic density [40–43], which in turn decreases the sensitivity of mammographic screening [44]. This hypothesis is supported by some studies [45, 46], but not by others [47, 48]. Another suggested explanation is that women using HT have faster-growing tumours, which will contribute to increased risk of interval cancer [49]. Increased medical surveillance in HT users has also been suggested but deemed unlikely as an explanation [47, 49].

To our knowledge, no previous studies have reported the risk of interval cancer according to route of administration of HT. Our results showed that use of any type of HT are more strongly associated with increased risk of interval cancer than screen-detected cancer. The risk of interval cancer is especially noticeable in users of oral (HR 2.46) and transdermal (HR 2.41) oestradiol.

There have been several advances and attempts over the years to develop HT preparations with a safer profile. Thus, despite the association between use of HT and BC risk is well known, it is important to keep monitoring the effects of these various preparations on BC risk to ensure that the information given to women is correct, to help them make an informed choice.

This is one of the largest studies on the association between the use of HT and risk of BC. The linkage of high-quality nationwide registries ensured detailed information on HT exposure, including the type of HT, route of administration and individual drugs used. The registry linkage avoided exposure recall bias and population selection bias, which leads to reliable estimates for the current use of HT as well as for past use. HT use was updated during follow-up, which has been shown to be important to avoid underestimation of the associations of interest [50]. We had information about important BC risk factors such as parity, BMI, and education, in addition to cancer information such as molecular subtype, detection mode, and stage.

There are however several limitations. First, we do not have any information about HT use prior to 2004, and therefore do not know the full duration of use among women using it at baseline, or even in the first few years after 2004, as many women stop using HT, sometimes for several years, before continuing. In addition, it will lead to some misclassification of past users as non-users, which would result in underestimated associations. Second, we do not know the women’s compliance, thus some misclassification of non-/past users as current users is expected. It is, however, unlikely that women continue to redeem HT prescriptions from the pharmacy without using it, and therefore the misclassified person time as current users should be small, while the risk associated with past HT use might be underestimated. We further expect some misclassification of the duration of current use as we do not know the exact dose each woman used (i.e., whether she took the medication daily), and thereby do not know the precise duration of each prescription. Third, we did not have information on menopausal status, a potentially confounding factor. In a sensitivity analysis with restricted follow-up from age 55 the conclusions remained the same. However, the hazard ratios increased, therefore the estimates we present in this study are likely conservative. Fourth, we did not have information about breast cancer gene (BRCA)1/2 mutations. This is not a contraindication for HT in Norway, however women with these mutations are probably less inclined to use HT, which could lead to underestimation of associations. Fifth, we also lacked information about hysterectomy, which is a potential confounder. Finally, the HT users are likely representative of women who experience severe menopausal symptoms, while the non-users likely represent women without severe symptoms, thus generalising the results to the general female population should be done with some caution.

In conclusion, oral and transdermal use of HT was associated with increased risk of BC, while use of vaginal HT was not. The highest risk was found among oral oestradiol-NETA users, and among women with low BMI. Use of HT was more strongly associated with the risk of luminal A BC than the other BC subtypes, and more strongly associated with the risk of interval cancer than screen-detected cancer. We found indication for a lower risk associated with the use of the newer oral oestradiol-NETA product Cliovelle®, but this needs to be confirmed in more tailored studies.

## Disclaimer

The Norwegian Institute of Public Health is not responsible for the content of publications, analyses or conclusions based on the provided data. The interpretation and reporting of these data are the sole responsibility of the authors, and no endorsement by the Cancer Registry of Norway is intended nor should be inferred. Where authors are identified as personnel of the International Agency for Research on Cancer/WHO, the authors alone are responsible for the views expressed in this article, and they do not necessarily represent the decisions, policy, or views of the International Agency for Research on Cancer/WHO.

## Supplementary information

Supplementary material for Menopausal hormone therapy and breast cancer risk: a population-based cohort study of 1,3 million women in Norway

## Data Availability

Research data used in the analyses can be made available on request to https://helsedata.no/, given the legal basis in Articles 6 and 9 of the GDPR and that the processing is in accordance with Article 5 of the GDPR, with additional national legal basis as per the Regulations on population-based health surveys.
