# Correction: Menopausal hormone therapy and breast cancer risk: a population-based cohort study of 1.3 million women in Norway

**DOI:** 10.1038/s41416-025-03038-w

**Published:** 2025-08-20

**Authors:** Nathalie C. Støer, Siri Vangen, Deependra Singh, Renée T. Fortner, Solveig Hofvind, Giske Ursin, Edoardo Botteri

**Affiliations:** 1 Department of Research, Cancer Registry of Norway, Norwegian Institute of Public Health, Oslo, Norway; 2 Norwegian Research Centre for Women’s Health, Division of Obstetrics and Gynecology, Oslo University Hospital, Oslo, Norway; 3 Institute of Clinical Medicine, University of Oslo, Oslo, Norway; 4 Cancer Surveillance Branch, International Agency for Research on Cancer, WHO, Lyon, France; 5 Division of Cancer Epidemiology, German Cancer Research Center (DKFZ), Heidelberg, Germany; 6 Section for Breast Cancer Screening, Cancer Registry of Norway, Norwegian Institute of Public Health, Oslo, Norway; 7 Department of Health and Care Sciences, UiT The Arctic University of Norway, Tromsø, Norway; 8 Cancer Registry of Norway, Norwegian Institute of Public Health, Oslo, Norway; 9 Department of Nutrition, Institute of Basic Medical Sciences, University of Oslo, Oslo, Norway; 10 Department of Preventive Medicine, University of Southern California, Los Angeles, CA USA; 11 Section for Colorectal Cancer Screening, Cancer Registry of Norway, Norwegian Institute of Public Health, Oslo, Norway

**Keywords:** Risk factors, Breast cancer

Correction to: *British Journal of Cancer* 10.1038/s41416-024-02590-1, published online 13 May 2024

Following publication of the article, the authors identified an error in the variable defining breast cancer detection mode; this affected the data presented in Table 5. The table has been corrected to rectify the incorrect number of screening detected cancers, interval cancers and cancers detected outside of the screening program, and to provide correct estimates of the association between the use of menopausal hormone therapy and risk of interval cancers, screening detected cancers and cancers detected outside of the screening program.

**Table 5:** Use of menopausal hormone therapy and risk of breast cancer stratified by detection mode among 925,874 women aged 50-71 (women eligible for mammographic screening with two years of additional follow-up).

	Interval cancer		Screening detected cancer		Detected outside of screening		p_heterogeneity_
	Cases	HR (95% CI)	Cases	HR (95% CI)	Cases	HR (95% CI)	
No use	2,036	Ref.	7,073	Ref.	3,035	Ref.	
Current HT	1,206	2.00 (1.85 – 2.15)	2,857	1.40 (1.34 – 1.47)	1,064	1.30 (1.20 – 1.39)	<0.001
Past HT	817	1.24 (1.14 – 1.35)	2,361	1.02 (0.98 – 1.08)	849	0.90 (0.83 – 0.97)	<0.001
Type of component in oral or transdermal current users^b^							
Oestradiol	137	2.49 (2.09 – 2.96)	216	1.16 (1.00 – 1.32)	85	1.12 (0.91 – 1.39)	<0.001
Oestriol^a^	15	1.25 (0.80 – 2.08)	41	0.93 (0.68 – 1.26)	19	0.95 (0.60 – 1.49)	0.606
Oestradiol-NETA	478	2.89 (2.61 – 3.19)	1,181	2.16 (2.03 – 2.30)	453	2.01 (1.82 – 2.22)	<0.001
Tibolone^a^	78	2.50 (2.00 – 3.14)	141	1.39 (1.18 – 1.65)	79	1.93 (1.55 – 2.42)	<0.001
Route of administration in oral or transdermal current users^c^							
Oral oestradiol	88	2.46 (1.99 – 3.05)	133	1.07 (0.90 – 1.28)	53	1.05 (0.80 – 1.37)	<0.001
Transdermal oestradiol	43	2.41 (1.78 – 3.26)	74	1.28 (1.01 – 1.60)	27	1.17 (0.80 – 1.71)	0.002
Oral oestradiol-NETA	410	2.89 (2.60 – 3.22)	1,049	2.21(2.07 – 2.36)	387	1.98 (1.78 – 2.21)	<0.001
Transdermal oestradiol-NETA	8	1.91 (0.95 – 3.83)	25	1.91(1.29 – 2.83)	4		1.000
Type of component in vaginal current users^d^							
Vaginal oestradiol	309	1.18 (1.04 – 1.33)	853	0.96 (0.89 – 1.03)	250	0.71 (0.62 – 0.81)	<0.001
Vaginal oestriol	6	1.26 (0.57 – 2.82	15	0.89 (0.54 – 1.48)	2		0.470

Hazard ratios (HRs) and 95% confidence intervals (CIs) from Cox regression with age as time scale (age adjusted) and additionally adjusted for ethnicity, number of children, education, income, health region, screening attendance (never, <2.5 years since last screening, ≥2.5 years since last screening), use of antidiabetics (A10), antithrombotic agents (B01), antihypertensives (C02), diuretics (C03), beta-blockers (C07), calcium channel blockers (C08), angiotensin-converting enzyme inhibitors and angiotensin receptor blockers (C09), lipid modifying agents (C10), uterotonics and other gynecologicals (G02), urologicals (G04), thyroid therapy (H03) and treatment of bone diseases (M05). HT: hormone therapy; NETA: norethisterone acetate. ^b,c,d^Estimates for mixed use not shown (^b^Interval cancer: 177 cases, screening detected cancer: 395 cases, detected outside of screening: 169 cases ^c^Interval cancer: total mixed use: 248 cases, mixed use due to switch between oral and transdermal oestradiol 6, mixed use due to switch between oral and transdermal oestradiol-NETA: 60, screening detected cancer: total mixed use 512 cases, mixed use due to switch between oral and transdermal oestradiol 9 cases, mixed use due to switch between oral and transdermal oestradiol-NETA 107 cases, detected outside of screening: total mixed use: 240 cases, mixed use due to switch between oral and transdermal oestradiol: 5 cases, mixed use due to switch between oral and transdermal oestradiol-NETA: 62 cases, ^d^ Interval cancer: total mixed use: 240 cases, mixed use due to switch between vaginal oestradiol and oestriol: 5 cases, screening detected cancer: total mixed use: 495 cases, mixed use due to switch between vaginal oestradiol and oestriol: 5 cases, detected outside of screening: total mixed use: 232 cases, mixed use due switch between vaginal oestradiol and oestriol: 4 cases).

The corrected version of Table 5 is presented below.

The corrected data impacts three sections of the article; the original and corrected paragraphs can be found below, with the affected areas marked in bold.

The corrections do not affect the conclusions of the article. The article has been updated.

Original abstract:

Results: We included 1,275,783 women, 45+ years, followed from 2004, for a median of 12.7 years. Oral oestrogen combined with daily progestin was associated with the highest risk of BC (HR 2.42, 95% confidence interval (CI) 2.31–2.54), with drug-specific HRs ranging from Cliovelle®: 1.63 (95% CI 1.35–1.96) to Kliogest®: 2.67 (2.37–3.00). Vaginal oestradiol was not associated with BC risk. HT use was more strongly associated with luminal A cancer (HR 1.97, 95% CI 1.86–2.09) than other molecular subtypes, and more strongly with interval (HR 2.00, 95% **CI: 1.83–2.30**) than screen-detected (**HR 1.33, 95% CI 1.26–1.41**) BC in women 50–71 years. HRs for HT use decreased with increasing BMI.

Corrected abstract:

Results: We included 1,275,783 women, 45+ years, followed from 2004, for a median of 12.7 years. Oral oestrogen combined with daily progestin was associated with the highest risk of BC (HR 2.42, 95% confidence interval (CI) 2.31–2.54), with drug-specific HRs ranging from Cliovelle®: 1.63 (95% CI 1.35–1.96) to Kliogest®: 2.67 (2.37–3.00). Vaginal oestradiol was not associated with BC risk. HT use was more strongly associated with luminal A cancer (HR 1.97, 95% CI 1.86–2.09) than other molecular subtypes, and more strongly with interval (HR 2.00, 95% **CI: 1.85–2.15**) than screen-detected (**HR 1.40, 95% CI 1.34–1.47**) BC in women 50–71 years. HRs for HT use decreased with increasing BMI.

Original Results paragraph:

Compared to HT non-users, current users of HT had a higher risk of interval cancer (HR 2.00, 95% **CI 1.83–2.30**) than screen-detected BC (**HR 1.33, 95% CI 1.26–1.41**), or cancer detected outside the screening programme (**HR 1.50, 95% CI 1.43–1.57**; P_heterogeneity_ < 0.001; Table 5). A similar pattern was observed across all exposure categories, with significant heterogeneity for past HT use (P_heterogeneity_ <0.001), and current use of oestradiol (P_heterogeneity_ overall <0.001, oral <0.001), oestradiol-NETA (P_heterogeneity_ overall <0.001, oral <0.001) and tibolone (P_heterogeneity_=**0.022**).

Corrected Results paragraph:

Compared to HT non-users, current users of HT had a higher risk of interval cancer (HR 2.00, 95% **CI 1.85–2.15**) than screen-detected BC (**HR 1.40, 95% CI 1.34–1.47**), or cancer detected outside the screening programme (**HR 1.30, 95% CI 1.20–1.39**; P_heterogeneity_ < 0.001; Table 5). A similar pattern was observed across all exposure categories, with significant heterogeneity for past HT use (P_heterogeneity_ <0.001), and current use of oestradiol (P_heterogeneity_ overall <0.001, oral <0.001), oestradiol-NETA (P_heterogeneity_ overall <0.001, oral <0.001) and tibolone (P_heterogeneity_<**0.001**).

Original Discussion paragraph:

To our knowledge, no previous studies have reported the risk of interval cancer according to route of administration of HT. Our results showed that use of any type of **non-vaginal HT** are more strongly associated with increased risk of interval cancer than screen-detected cancer. The risk of interval cancer is especially noticeable in users of oral (**HR 2.68**) and transdermal (**HR 1.89**) oestradiol.

Corrected Discussion paragraph:

To our knowledge, no previous studies have reported the risk of interval cancer according to route of administration of **HT**. Our results showed that use of any type of HT are more strongly associated with increased risk of interval cancer than screen-detected cancer. The risk of interval cancer is especially noticeable in users of oral (**HR 2.46**) and transdermal (**HR 2.41**) oestradiol.

The original article has been corrected.

