# Supplementary material for: Menopausal hormone therapy and breast cancer risk: a population-based cohort study of 1.3 million women in Norway
Source: Br J Cancer. 2024 May 13;131(1):126–37. doi: 10.1038/s41416-024-02590-1 (PMC11231299; doi:10.1038/s41416-024-02590-1)
Supplement: Supplementary file 1 — Supplementary material for Menopausal hormone therapy and breast cancer risk: a population-based cohort study of 1,3 million women in Norway [file 41416_2024_2590_MOESM1_ESM.pdf]

Supplementary material for *Menopausal hormone therapy and breast cancer risk: a population-based cohort study of 1,3 million women in Norway.*

Nathalie C. Støer, Siri Vangen, Deependra Singh, Renée T. Fortner, Solveig Hofvind, Giske Ursin, Edoardo Botteri

Page 2.	Supplementary table 1: Cohort characteristics
Page 4.	Supplementary table 2: Stratification by stage at diagnosis
Page 5.	Supplementary table 3: Sensitivity analysis among women with available body mass index
Page 6.	Supplementary table 4: Sensitivity analysis among women aged 55 or older
Page 7.	Supplementary table 5: Sensitivity analysis in the nested case-control sample
Page 8.	Supplementary table 6: Preparation specific estimates with no grouping by dose
Page 10.	Supplementary figure 1: Flow-chart of study population
Page 11.	Supplementary figure 2: Graphical description of follow-up and exposure definition
Page 12.	Supplementary figure 3: Dose-response analysis for time since last use of HT
Page 13.	Supplementary figure 4: Number of HT users per year in Norway

Supplementary table 1: Cohort characteristics according to use of menopausal hormone therapy

	**No HT (N=821,524)**	**Oestradiol only [a] (N=220,214)**	**Oestriol only [a] (N=29,444)**	**Tibolone only [a] (N=10,269)**	**Oestradiol-NETA only [a] (N=81,166)**	**Mixed use [a,b] (N=113,169)**
**Age in years** [c,d]	47.0 (45.0, 60.6)	53.3 (46.0, 60.8)	67.2 (57.0,74.1)	53.1 (45.5, 58.5)	48.4 (45.0, 56.2)	51.4 (45.0, 58.2)
**Ethnicity** [d], n = 1,275,786						
Norway	685,351 (83.4%)	199,641 (90.7%)	27,542 (93.5%)	9,189 (89.5%)	70,745 (87.2%)	101026 (89.3%)
Other Nordic	29,120 (3.5%)	6,411 (2.9%)	496 (1.7%)	370 (3.6%)	2,741 (3.4%)	3,456 (3.1%)
Non-Nordic	107,053 (13.0%)	14,162 (6.4%)	1,406 (4.8%)	710 (6.9%)	7,680 (9.5%)	8,687 (7.7%)
**Children** [d], n = 1,275,786						
0	124,428 (15.1%)	18,695 (8.5%)	3,067 (10.4%)	1,107 (10.8%)	9,380 (11.6%)	10,808 (9.6%)
1	111,678 (13.6%)	26,348 (12.0%)	3,498 (11.9%)	1,455 (14.2%)	12,296 (15.1%)	16,155 (14.3%)
2	299,981 (36.5%)	91,061 (41.4%)	9,656 (32.8%)	4,516 (44.0%)	33,747 (41.6%)	48,305 (42.7%)
3	192,823 (23.5%)	58,838 (26.7%)	7,836 (26.6%)	2,449 (23.8%)	19,081 (23.5%)	27,377 (24.2%)
≥4	92,614 (11.3%)	25,272 (11,5%)	5,387 (18.3%)	742 (7.2%)	6,662 (8.2%)	10,524 (9.3%)
**Education** [d], n = 1,231,472						
Non/mandatory only	226,608 (29.0%)	54,964 (25.2%)	12,273 (42.1%)	2,238 (22.0%)	21,904 (27.3%)	27,338 (24.3%)
Secondary	324,995 (41.6%)	100,792 (46.1%)	12,498 (42.9%)	4,712 (46.4%)	36,137 (45.1%)	50,875 (45.3%)
Higher	229,616 (29.4%)	62,727 (28.7%)	4,394 (15.1%)	3,202 (31.5%)	22,071 (27.6%)	34,128 (30.4%)
**Income** [d,e], n = 1,255,230						
Q1: <192,159 NOK	212,262 (26.5%)	48,700 (22.1%)	14,501 (49.4%)	1,618 (15.8%)	15,498 (19.1%)	21,229 (18.8%)
Q2: 192,159-259,606 NOK	187,140 (23.3%)	63,519 (28.9%)	7,814 (26.6%)	2,653 (25.9%)	22,539 (27.8%)	30,143 (26.6%)
Q3: 259,607-352,924 NOK	187,865 (23.4%)	61,963 (28.1%)	4,653 (15.8%)	3,205 (31.3%)	23,630 (29.2%)	32,492 (28.7%)
Q4: >352,924 NOK	214,004 (26.7%)	45,952 (20.9%)	2,414 (8.2%)	2,776 (27.1%)	19,383 (23.9%)	29,277 (25.9%)
**BMI** [f], n = 579,247						
<18.5	3,938 (1.3%)	1,398 (1.0%)	172 (1.4%)	86 (1.3%)	705 (1.5%)	776 (1.1%)
18.5-24.9	133,593 (45.6%)	70,765 (48.5%)	5,116 (42.2%)	3,479 (51.5%)	24,485 (51.0%)	37,793 (51.5%)
25.0-29.9	103,308 (35.3%)	53,016 (36.3%)	4,567 (37.7%)	2,354 (34.8%)	16,419 (34.2%)	26,039 (35.5%)
30.0-34.9	37,979 (13.0%)	16,080 (11.0%)	1,652 (13.6%)	688 (10.2%)	4,946 (10.3%)	6,990 (9.5%)
≥35.0	14,229 (4.9%)	4,760 (3.3%)	603 (5.0%)	154 (2.3%)	1,409 (2.9%)	1,748 (2.4%)
**Health region** [d], n= 1,244,853						
South-East	453,064 (57.1%)	120,127 (54.9%)	14,789 (50.4%)	6,074 (59.5%)	48,219 (59.8%)	63,707 (56.5%)
West	158,577 (20.0%)	37,641 (17.2%)	7,098 (24.2%)	1,927 (18.9%)	16,621 (20.6%)	21,394 (19.0%)
Mid	99,124 (12.5%)	39,719 (18.1%)	3,121 (10.6%)	991 (9.7%)	9,581 (11.9%)	16,541 (14.7%)
North	82,204 (10.4%)	21,484 (9.8%)	4,312 (14.7%)	1,208 (11.8%)	6,226 (7.7%)	11,121 (9.9%)

**Ever use A10** [a], n = 1,275,786	62,200 (7.6%)	17,163 (7.8%)	3,785 (12.9%)	570 (5.6%)	5,057 (6.2%)	7,989 (7.1%)
**Ever use B01** [a], n = 1,275,786	159,467 (19.4%)	61,532 (27.9%)	13,045 (44.3%)	2,285 (22.3%)	15,855 (19.5%)	29,993 (26.5%)
**Ever use C02** [a], n = 1,275,786	9,787 (1.2%)	2,969 (1.3%)	671 (2.3%)	127 (1.2%)	750 (0.9%)	1,340 (1.2%)
**Ever use C03** [a], n = 1,275,786	162,856 (19.8%)	53,345 (24.2%)	12,391 (42.1%)	2,122 (20.7%)	16,645 (20.5%)	26,659 (23.6%)
**Ever use C07** [a], n = 1,275,786	178,136 (21.7%)	61,513 (27.9%)	12,669 (43.0%)	2,447 (23.8%)	18,502 (22.8%)	31,103 (27.5%)
**Ever use C08** [a], n = 1,275,786	129,246 (15.7%)	46,034 (20.9%)	9,489 (32.2%)	1,735 (16.9%)	12,093 (14.9%)	21,216 (18.7%)
**Ever use C09** [a], n = 1,275,786	225,166 (27.4%)	78,399 (35.6%)	14,511 (49.3%)	3,349 (32.6%)	23,094 (28.5%)	36,439 (32.2 %)
**Ever use C10** [a], n = 1,275,786	201,568 (24.5%)	83,161 (37.8%)	14,109 (47.9%)	3,353 (32.7%)	21,927 (27.0%)	38,956 (34.4%)
**Ever use G02** [a], n = 1,275,786	107,237 (13.1%)	14,742 (6.7%)	635 (2.2%)	569 (5.5%)	5,817 (7.2%)	8,229 (7.3%)
**Ever use G04** [a], n = 1,275,786	43,140 (5.3%)	33,800 (15.3%)	5,855 (19.9%)	819 (8.0%)	6,762 (8.3%)	19,364 (17.1%)
**Ever use H03** [a], n = 1,275,786	96,197 (11.6%)	36,520 (16.6%)	5,460 (18.5%)	1,567 (15.3%)	11,919 (14.7%)	20,258 (17.9%)
**Ever use M05** [a], n = 1,275,786	58,721 (7.1%)	27,199 (12.4%)	5,782 (19.6%)	950 (9.3%)	5,833 (7.2%)	12,525 (11.1%)

HT: menopausal hormone therapy, NETA-norethisterone acetate, BMI: body mass index, A10: antidiabetics, B01: antithrombotic agents, C02: antihypertensives, C03: diuretics, C07: beta-blockers, C08: calcium channel blockers, C09: angiotensin-converting enzyme inhibitors and angiotensin receptor blockers, C10: lipid modifying agents, G02: uterotonics and other gynecologicals, G04 – urologicals, H03: thyroid therapy, M05: treatment of bone diseases.

[a] prescribed anytime during follow-up; [b] including medroxyprogestereone-NETA; [c] median (interquartile range); [d] registered at baseline; [e] income after tax per household unit; [f] measurement closest to baseline

Supplementary table 2: Use of menopausal hormone therapy and risk of breast cancer stratified by stage at diagnosis.

	Localized		Regionally advanced		Metastatic	
	**Cases**	**HR (95% CI)**	**Cases**	**HR (95% CI)**	**Cases**	**HR (95% CI)**
No use	11,956	Ref.	6,828	Ref.	949	Ref.
Current HT	4,272	1.53 (1.47 – 1.58)	1,876	1.39 (1.32 – 1.47)	150	0.91 (0.76 – 1.09)
Past HT	3,654	1.08 (1.04 – 1.13)	1,619	0.98 (0.93 – 1.04)	146	0.67 (0.56 – 0.80)
**Type of component in oral or transdermal current users**						
Oestradiol	341	1.37 (1.23 – 1.53)	188	1.53 (1.32 – 1.77)	21	1.46 (0.94 – 2.25)
Oestriol[a]	113	1.05 (0.87 – 1.27)	66	1.07 (0.84 – 1.37)	10	0.90 (0.48 – 1.69)
Oestradiol-NETA	1,649	2.38 (2.26 – 2.51)	705	2.05 (1.89 – 2.22)	62	1.52 (1.17 – 1.97)
Tibolone[a]	205	1.65 (1.44 – 1.90)	108	1.86 (1.54 – 2.25)	8	1.17 (0.58 – 2.35)
**Route of administration in current users**						
Oral oestradiol	213	1.29 (1.13 – 1.48)	125	1.54 (1.29 – 1.84)	12	1.20 (0.68 – 2.13)
Transdermal oestradiol	110	1.43 (1.18 – 1.72)	59	1.54 (1.19 – 2.00)	7	1.72 (0.82 – 3.63)
Oral oestradiol-NETA	1,460	2.43 (2.30 – 2.56)	613	2.06 (1.89 – 2.24)	56	1.57 (1.19 – 2.06)
Transdermal oestradiol-NETA	31	1.84 (1.29 – 2.62)	11	1.31 (0.72 – 2.36)	0	
**Type of component in vaginal current users**						
Vaginal oestradiol	1,302	1.03 (0.97 – 1.09)	528	0.91 (0.83 – 1.00)	33	0.47 (0.33 – 0.67)
Vaginal oestriol	27	0.87 (0.60 – 1.27)	16	0.94 (0.57 – 1.53)	2	

Hazard ratios (HRs) and 95% confidence intervals (CIs) from Cox regression with age as time scale (age adjusted) and additionally adjusted for ethnicity, number of children, education, income, health region, screening attendance (never, <2.5 years since last screening, ≥2.5 years since last screening), use of antidiabetics (A10), antithrombotic agents (B01), antihypertensives (C02), diuretics (C03), beta-blockers (C07), calcium channel blockers (C08), angiotensin-converting enzyme inhibitors and angiotensin receptor blockers (C09), lipid modifying agents (C10), uterotonics and other gynecologicals (G02), urologicals (G04), thyroid therapy (H03) and treatment of bone diseases (M05). Estimates for mixed use not shown. HT: hormone therapy; NETA: norethisterone acetate. [a]Oral formulation.

Supplementary table 3: Use of menopausal hormone therapy and risk of breast cancer among 579,247 women with available information on body mass index.

	Cases	Person years	Model 1: HR (95% CI)	Model 2: HR (95% CI)
No use	9,751	4,752,074	Ref.	Ref.
Current HT	4,159	1,245,428	1.40 (1.35 – 1.45)	1.40 (1.35 – 1.46)
Past HT	3,807	1,432,149	1.07 (1.03 – 1.11)	1.07 (1.03 – 1.11)
**Type of component in oral or transdermal current users**				
Oestradiol	349	109,793	1.40 (1.26 – 1.56)	1.40 (1.26 – 1.56)
Oestriol[a]	78	27,259	1.11 (0.89 – 1.38)	1.11 (0.89 – 1.38)
Oestradiol-NETA	1,551	329,095	2.16 (2.05 – 2.28)	2.17 (2.06 – 2.30)
Oestradiol-MPA	14	2,986	2.04 (1.21 – 3.45)	2.04 (1.21 – 3.45)
Tibolone[a]	195	57,702	1.46 (1.27 – 1.68)	1.46 (1.27 – 1.69)
**Type of combined EPT regimen in current users**				
Continuous oestradiol-NETA	1,236	236,934	2.26 (2.13 – 2.40)	2.27 (2.14 – 2.41)
Sequential oestradiol-NETA	143	55,191	1.51 (1.28 – 1.78)	1.51 (1.28 – 1.79)
**Route of administration in oral or transdermal current users**				
Oral oestradiol	220	73,335	1.30 (1.14 – 1.49)	1.30 (1.14 – 1.49)
Transdermal oestradiol	112	33,474	1.53 (1.27 – 1.84)	1.53 (1.27 – 1.84)
Oral continuous oestradiol-NETA	1,206	228,962	2.28 (2.15 – 2.42)	2.36 (2.15 – 2.43)
Oral sequential oestradiol-NETA	136	53,089	1.50 (1.26 – 1.78)	1.50 (1.27 – 1.78)
Continuous transdermal oestradiol-NETA	23	6,008	1.71 (1.13 – 2.57)	1.72 (1.14 – 2.58)
Sequential transdermal oestradiol-NETA	5	1,652	1.62 (0.68 – 3.90)	1.63 (0.68 – 3.91)
**Type of component in vaginal current users**				
Vaginal oestradiol	1,323	553,935	0.93 (0.88 – 0.99)	0.93 (0.88 – 0.99)
Vaginal oestriol	25	11,487	0.91 (0.62 – 1.35)	0.91 (0.62 – 1.35)

Hazard ratios (HRs) and 95% confidence intervals (CIs) from Cox regression with age as time scale (age adjusted) and adjusted for ethnicity, number of children, education, income, health region, screening attendance (never, <2.5 years since last screening, ≥2.5 years since last screening), use of antidiabetics (A10), antithrombotic agents (B01), antihypertensives (C02), diuretics (C03), beta-blockers (C07), calcium channel blockers (C08), angiotensin-converting enzyme inhibitors and angiotensin receptor blockers (C09), lipid modifying agents (C10), uterotonics and other gynecologicals (G02), urologicals (G04), thyroid therapy (H03) and treatment of bone diseases (M05) (Model 1), additionally adjusted for body mass index (Model 2). Estimates for mixed use not shown. HT: hormone therapy; NETA: norethisterone acetate; MPA: medroxyprogesterone acetate. [a]Oral formulation.

Supplementary table 4: Use of menopausal hormone therapy and risk of breast cancer among women aged 55 or older.

	Cases	Person years	HR (95% CI)
No use	13,353	5,385,609	Ref.
Current HT	5,619	1,355,668	1.56 (1.51 – 1.61)
Past HT	5,235	1,812,753	1.09 (1.05 – 1.12)
**Type of component in oral or transdermal current users**			
Oestradiol	429	99,583	1.62 (1.47 – 1.78)
Oestriol[a]	215	71,553	1.14 (0.99 – 1.30)
Oestradiol-NETA	2,015	284,683	2.75 (2.62 – 2.89)
Oestradiol-MPA[a]	14	2,786	1.98 (1.17 – 3.34)
Tibolone[a]	286	56,157	1.93 (1.71 – 2.17)
**Type of combined EPT regimen in current users**			
Continuous oestradiol-NETA	1,756	240,015	2.81 (2.67 – 2.95)
Sequential oestradiol-NETA	97	19,684	2.06 (1.69 – 2.52)
**Route of administration in oral or transdermal current users**			
Oral oestradiol	296	70,805	1.56 (1.39 – 1.75)
Transdermal oestradiol	118	26,698	1.69 (1.41 – 2.02)
Oral oestriol	215	71,553	1.14 (0.99 – 1.30)
Oral continuous oestradiol-NETA	1,719	233,242	2.83 (2.69 – 2.98)
Oral sequential oestradiol-NETA	93	18,775	2.08 (1.69 – 2.55)
Continuous transdermal oestradiol-NETA	27	5,137	2.03 (1.39 – 2.96)
Sequential transdermal oestradiol-NETA	4	808	
**Type of component in vaginal current users**			
Vaginal oestradiol	1,790	660,836	1.01 (0.96 – 1.06)
Vaginal oestriol	48	19,146	0.98 (0.74 – 1.30)

Hazard ratios (HRs) and 95% confidence intervals (CIs) from Cox regression with age as time scale (age adjusted) and adjusted for ethnicity, number of children, education, income, health region, screening attendance (never, <2.5 years since last screening, ≥2.5 years since last screening), use of antidiabetics (A10), antithrombotic agents (B01), antihypertensives (C02), diuretics (C03), beta-blockers (C07), calcium channel blockers (C08), angiotensin-converting enzyme inhibitors and angiotensin receptor blockers (C09), lipid modifying agents (C10), uterotonics and other gynecologicals (G02), urologicals (G04), thyroid therapy (H03) and treatment of bone diseases (M05). Estimates for mixed use not shown. HT: hormone therapy; NETA: norethisterone acetate; MPA: medroxyprogesterone acetate. [a]Oral formulation.

Supplementary table 5: Use of menopausal hormone therapy and risk of breast cancer in the nested case-control sample.

	**Cases**	**HR (95% CI)**
No use	21,221	Ref.
Current use	6,633	1.54 (1.50 – 1.59)
Past use	5,800	1.01 (0.98 – 1.05)
**Type of component in oral or transdermal current users**		
Oestradiol	513	1.45 (1.32 – 1.60)
Oestriol[a]	181	1.47 (1.25 – 1.72)
Oestradiol-NETA	2,340	2.31 (2.21 – 2.43)
Tibolone[a]	277	1.84 (1.62 – 2.10)
**Type of combined EPT regimen in current users**		
Continuous oestradiol-NETA	1,624	2.77 (2.62 – 2.94)
Sequential oestradiol-NETA	207	1.36 (1.17 – 1.57)
**Route of administration in oral or transdermal current users**		
Oral oestradiol	321	1.36 (1.21 – 1.53)
Transdermal oestradiol	158	1.55 (1.31 – 1.83)
Oral continuous oestradiol-NETA	1,678	2.71 (2.60 – 2.87)
Oral sequential oestradiol-NETA	254	1.34 (1.17 – 1.53)
Continuous transdermal oestradiol-NETA	20	1.74 (1.08 – 2.81)
Sequential transdermal oestradiol-NETA	6	0.96 (0.41 – 2.23)
**Type of component in vaginal current users**		
Vaginal oestradiol	1,378	0.99 (0.93 – 1.05)
Vaginal oestriol	34	1.03 (0.72 – 1.47)

Hazard ratios (HRs) and 95% confidence intervals (CIs) from stratified Cox regression adjusted for ethnicity, number of children, education, income, health region, screening attendance (never, <2.5 years since last screening, ≥2.5 years screening since last), use of antidiabetics (A10), antithrombotic agents (B01), antihypertensives (C02), diuretics (C03), beta-blockers (C07), calcium channel blockers (C08), angiotensin-converting enzyme inhibitors and angiotensin receptor blockers (C09), lipid modifying agents (C10), uterotonics and other gynecologicals (G02), urologicals (G04), thyroid therapy (H03) and treatment of bone diseases (M05). Estimates for mixed use not shown. HT: hormone therapy; NETA: norethisterone acetate. [a]Oral formulation.

Supplementary table 6: Use of menopausal hormone therapy preparations and risk of breast cancer with no grouping by dose.

	Strength	Cases	Person years	HR (95% CI)
No use		21,221	9,261,670	Ref.
**Oral oestradiol**				
Progynova®	1 mg oestradiol/day	140	38,319	1.39 (1.18 – 1.64)
Progynova®	2 mg oestradiol/day	162	46,378	1.40 (1.20 – 1.63)
**Transdermal oestradiol**				
Estradot®	0.025 mg oestradiol/day	12	4,377	1.11 (0.63 – 1.95)
Estradot®	0.0375 mg oestradiol/day	16	4,180	1.53 (0.94 – 2.50)
Estradot®	0.050 mg oestradiol/day	48	13,436.0	1.45 (1.09 – 1.92)
Estradot®	0.075 mg oestradiol/day	5	1,478	1.40 (0.58 – 3.38)
Estradot®	0.100 mg oestradiol/day	5	1,478	1.38 (0.58 – 3.32)
Evorel®	0.025 mg oestradiol/day	3	1,223	
Evorel®	0.050 mg oestradiol/day	7	2,400	1.18 (0.56 – 2.48)
Evorel®	0.100 mg oestradiol/day	1	436	
**Vaginal oestradiol**				
Vagifem® vaginal inserts	0.010 mg oestradiol/day	1,059	398,208	0.97 (0.91 – 1.03)
Vagifem® vaginal inserts	0.025 mg oestradiol/day	671	266,187	0.96 (0.89 – 1.04)
**Oral oestriol**				
Ovesterin®	1 mg oestriol/day	100	36,853	1.01 (0.83 – 1.23)
Ovesterin®	2 mg oestriol/day	45	12,559	1.34 (1.00 – 1.79)
**Vaginal oestriol**				
Ovesterin® vaginal inserts	0.5 mg oestriol/day	31	13,854	0.88 (0.62 – 1.26)
Ovesterin® vaginal cream	1 mg oestriol/1 g cream	20	6,924	1.13 (0.73 – 1.76)
**Oral continuous oestradiol-NETA**				
Eviana®	0.5 mg oestradiol/day; 2.8 mg NETA/month	72	16,922	1.66 (1.32 – 2.09)
Activelle®*	1 mg oestradiol /day; 14 mg NETA/month	957	155,051	2.42 (2.26 – 2.58)
Cliovelle®	1 mg oestradiol /day; 14 mg NETA/month	12	27,602	1.63 (1.35 – 1.96)
Kliogest®	2 mg oestradiol/day; 28 mg NETA/month	286	42,499	2.67 (2.37 – 3.00)
**Oral sequential oestradiol-NETA**				
Trisekvens®	2 mg oestradiol /day; 10 mg NETA/month	144	42,712	1.56 (1.32 – 1.83)
Trisekvens forte®	4 mg oestradiol /day; 10 mg NETA/month	3	939	
Novofem®	1 mg oestradiol /day; 12 mg NETA/month	84	31,936	1.23 (0.99 – 1.53)
**Transdermal continuous oestradiol-NETA**				
Estalis®	0.05 mg oestradiol /day; 7 mg NETA/month	34	8,649	1.57 (1.12 – 2.20)

**Transdermal sequential oestradiol-NETA**				
Sequidot®	0.05 mg oestradiol /day; 3.5 mg NETA/month	7	1,164	2.62 (1.25 – 5.50)
**Oral continuous oestradiol-MPA**				
Indivina®	1 mg oestriol/day; 2.8 mg MPA/month	9	2,066	1.73 (0.90 – 3.33)
Indivina®	1 mg oestriol/day; 5 mg MPA/month	2	464	
Indivina®	2 mg oestriol/day; 5 mg MPA/month	2	711	

Hazard ratios (HRs) and 95% confidence intervals (CIs) from Cox regression with age as time scale (age adjusted) and additionally adjusted for ethnicity, number of children, education, income, health region, screening attendance (never, <2.5 years since last screening, ≥ 2.5 years since last screening), use of antidiabetics (A10), antithrombotic agents (B01), antihypertensives (C02), diuretics (C03), beta-blockers (C07), calcium channel blockers (C08), angiotensin-converting enzyme inhibitors and angiotensin receptor blockers (C09), lipid modifying agents (C10), uterotonics and other gynecologicals (G02), urologicals (G04), thyroid therapy (H03) and treatment of bone diseases (M05). Estimates for mixed use not shown. NETA: norethisterone acetate; MPA: medroxyprogesterone acetate.

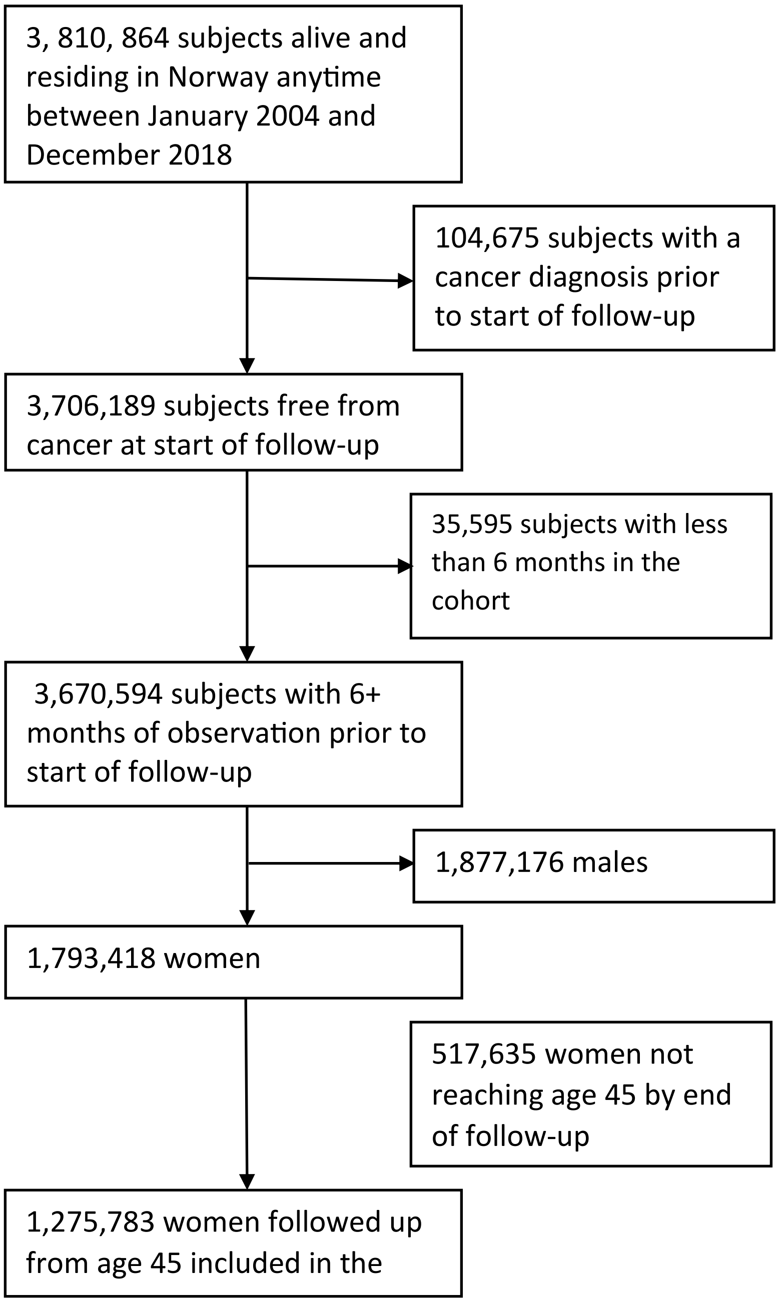

Supplementary figure 1. Flow-chart of study population.

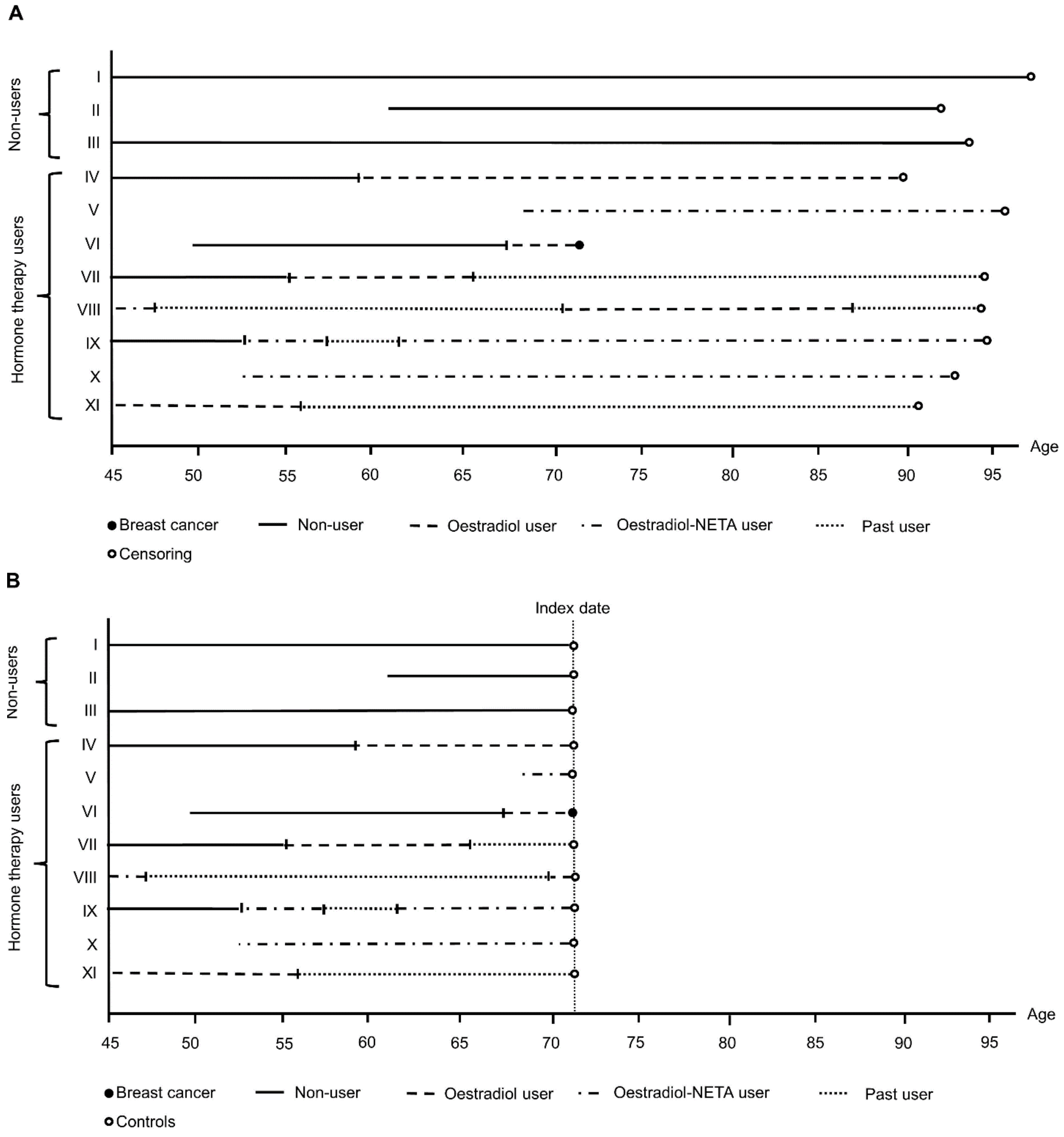

Supplementary figure 2. Graphical description of follow-up and exposure definition (only oestradiol and oestradiol-NETA for simplicity) in the cohort (A) and nested case-control sample (B). Follow-up starts 6 months after cohort entry.

A: Woman VI is diagnosed with breast cancer at age 72, the rest are censored. Woman I, III, IV, VII-IX and XI are included in the cohort at age 45, while the rest are included at an older age. Woman I-III are unexposed. Woman V, VIII, X and XI are exposed from start of follow-up, while woman IV, VI, VII and IX start using hormone therapy (HT) during follow-up. Woman VII, VIII, IX and XI stop using HT during follow-up and become past users, and woman VIII continue HT use

after a period of past use.
B: Woman VI is diagnosed with breast cancer at age 72, the rest are sampled as controls for woman VI. Duration of use was calculated for current users at index date, i.e. for woman IV-VI and VIII-X. Woman IV, VI and IX start using HT more than five years after cohort entry and their duration of use is the sum of the duration of all user periods until index date. Woman V, VIII, X and XI use HT in the five first years after cohort entry, i.e. defined as prevalent users. Among them, woman V and VIII have a total observed duration of less than five years and are thus categorized with unknown duration of use, while woman X and XI have a total observed duration of more than five years and are categorized with ≥5 years duration of use. Time since last use was calculated among past users at index date, i.e. for woman VII and XI.

	Cases	HR (95% CI)
**Oral ET**		
<1 year	71	0.91(0.71-1.16)
1- 2.9 years	98	0.95(0.77-1.17)
3 - 4.9 years	75	0.87(0.68-1.10)
5 - 9.9 years	143	0.86(0.72-1.02)
≥10 years	45	0.77(0.57-1.05)
**Vaginal ET**		
<1 year	570	0.94(0.86-1.02)
1- 2.9 years	666	0.96(0.89-1.04)
3 - 4.9 years	451	0.98(0.89-1.08)
5 - 9.9 years	522	0.89(0.81-0.97)
≥10 years	105	0.86(0.71-1.06)
**Oral oestrogen-NETA**		
<1 year	269	1.18(1.04-1.35)
1- 2.9 years	320	1.12(1.00-1.26)
3 - 4.9 years	237	1.08(0.94-1.24)
5 - 9.9 years	425	1.19(1.07-1.32)
≥10 years	155	1.20(1.01-1.42)
**Oral tibolone**		
<1 year	41	1.14(0.82-1.58)
1- 2.9 years	61	1.18(0.90-1.54)
3 - 4.9 years	39	1.05(0.75-1.46)
5 - 9.9 years	87	1.23(0.99-1.54)
≥10 years	25	1.00(0.66-1.51)

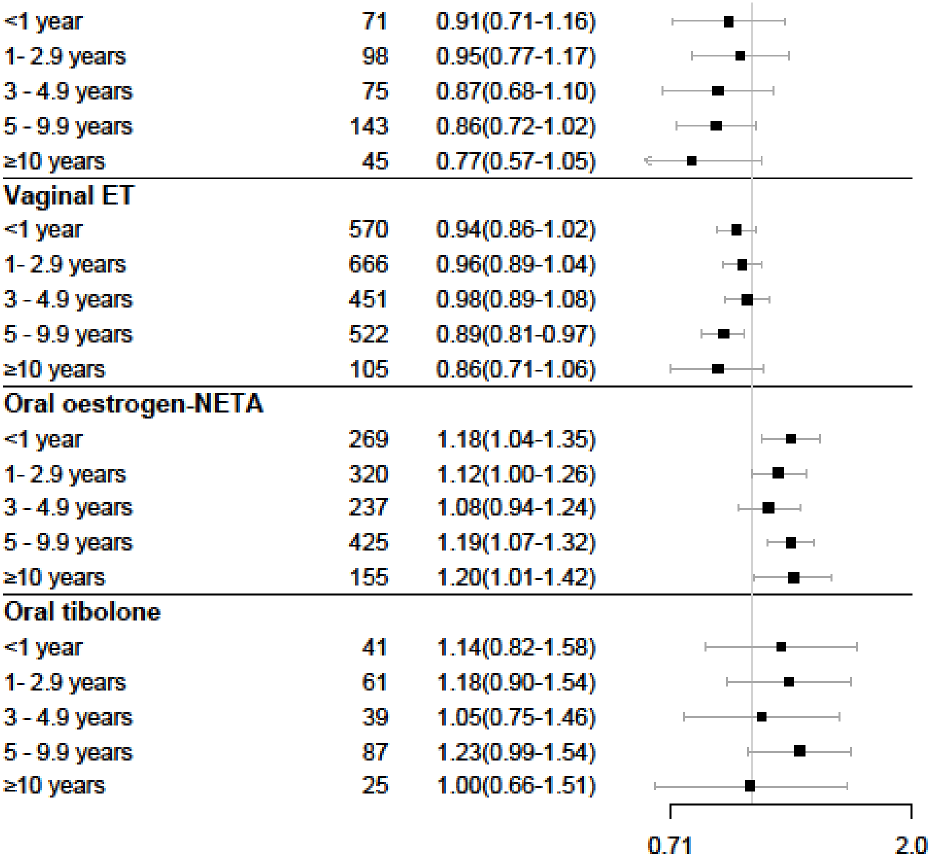

Supplementary figure 3. Use of menopausal hormone therapy and risk of breast cancer according to time since last use in past users compared to non-users in a 1:10 nested case-control sample. Hazard ratios (HRs) and 95% confidence intervals (CIs) from stratified Cox regression and additionally adjusted for ethnicity, number of children, education, income, health region, screening attendance (never, <2.5 years since last screening, ≥2.5 years since last

screening), use of antidiabetics (A10), antithrombotic agents (B01), antihypertensives (C02), diuretics (C03), beta-blockers (C07), calcium channel blockers (C08), angiotensin-converting enzyme inhibitors and angiotensin receptor blockers (C09), lipid modifying agents (C10), uterotonics and other gynecologicals (G02), urologicals (G04), thyroid therapy (H03) and treatment of bone diseases (M05). Estimates for mixed use not shown. ET:estrogen; NETA-norethisterone acetate.

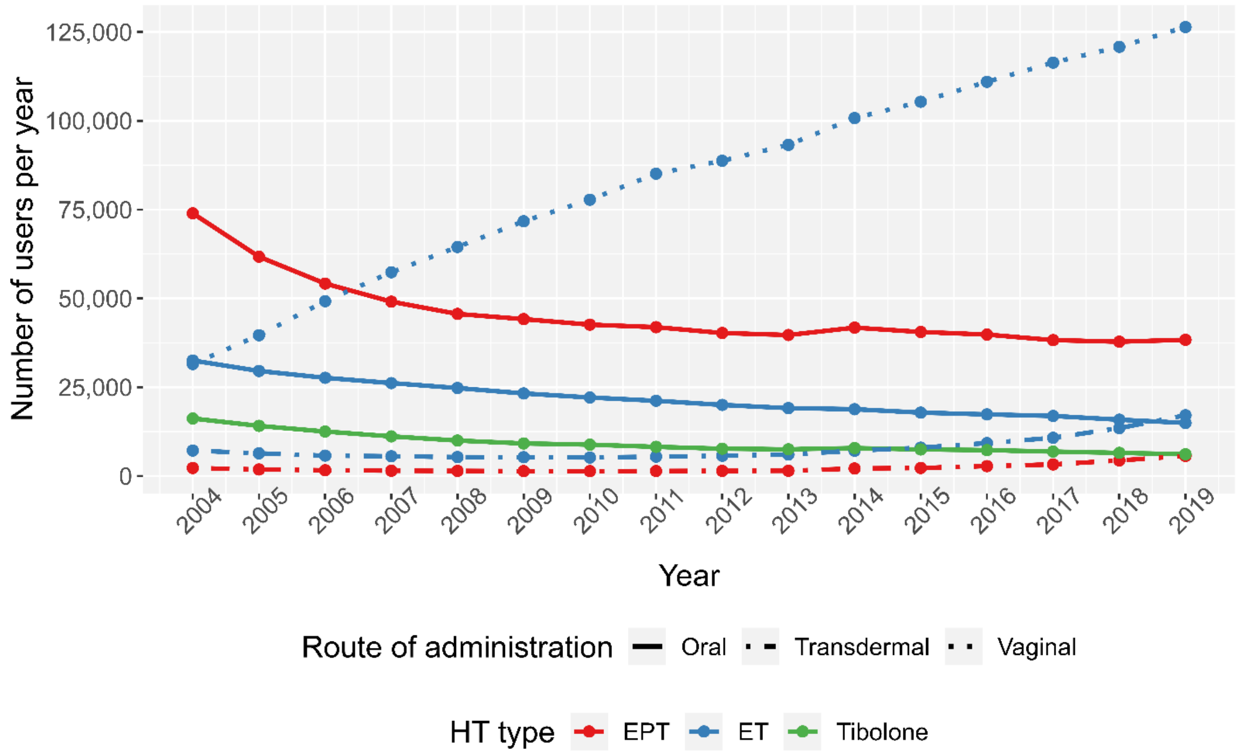

Supplementary figure 4: Number of menopausal hormone therapy users per year in Norway
